# Connexin 43 Hemichannels Regulate the Expression of Wound Healing-Associated Genes in Human Gingival Fibroblasts

**DOI:** 10.1038/s41598-017-12672-1

**Published:** 2017-10-26

**Authors:** Rana Tarzemany, Guoqiao Jiang, Jean X. Jiang, Hannu Larjava, Lari Häkkinen

**Affiliations:** 1 Department of Oral Biological and Medical Sciences, Faculty of Dentistry, The University of British Columbia, Vancouver, BC V6T 1Z3 Canada; 2 Department of Biochemistry, University of Texas Health Science Center, San Antonio, Texas 78229–3900 USA

## Abstract

Connexin 43 (Cx43) is the most ubiquitous connexin in various cells, and presents as hemichannels (HCs) and gap junctions (GJs) on the cell membrane. We have recently shown that Cx43 abundance was strongly reduced in fibroblasts of human gingival wounds, and blocking Cx43 function in cultured human gingival fibroblasts (GFBLs) strongly regulated the expression of wound healing-related genes. However, it is not known whether these responses involved Cx43 HCs or GJs. Here we show that Cx43 assembled into distinct GJ and HC plaques in GFBLs both *in vivo* and *in vitro*. Specific blockage of Cx43 HC function by TAT-Gap19, a Cx43 mimetic peptide, significantly upregulated the expression of several MMPs, TGF-β signaling molecules, Tenascin-﻿C, and VEGF-A, while pro-fibrotic molecules, including several extracellular matrix proteins and myofibroblast and cell contractility-related molecules, were significantly downregulated. These changes were linked with TAT-Gap19-induced suppression of ATP signaling and activation of the ERK1/2 signaling pathway. Collectively, our data suggest that reduced Cx43 HC function could promote fast and scarless gingival wound healing. Thus, selective suppression of Cx43 HCs may provide a novel target to modulate wound healing.

## Introduction

Connexins (Cxs) are a family of 21 molecules in humans that form hemichannels (HCs) and gap junctions (GJs) on the cell membrane. Each Cx molecule is composed of four transmembrane domains, two extracellular loops (E1 and E2), a cytoplasmic N-terminus, a cytoplasmic loop, and a C-terminal domain critical for the regulation of Cx function^1,2^. When present as HCs, Cxs form unopposed channels between cells and the extracellular space. HC opening can be induced by factors associated with, for example, wound healing, inflammation, hypoxia, oxidative stress, mechanical signals, changes in intra- and extracellular osmolarity and Ca^2+^ concentration, and cytosolic pH^2–5^. This provides a pathway for the transfer of small ions, metabolites, and signaling molecules between the cytosol and the extracellular space that regulate cell functions via auto- and paracrine mechanisms and by changing the intracellular milieu^1,2,6^. For instance, ATP, which has an intracellular concentration several-fold higher than the outside, moves out via HCs to interact with purinergic receptors in order to elicit auto- or paracrine signaling^1,7^. Collectively, HCs are involved in the regulation of various cell functions including survival, proliferation, migration, oxidative stress and gene expression^3^.

When Cxs form GJs, two HCs from opposing cells connect across the intercellular space, forming a channel that links the cytoplasms of the participating cells. This process is regulated by phosphorylation of Cxs at specific cytoplasmic sites, and interactions with cytoplasmic scaffolding proteins. Typically, several GJs cluster laterally to form large (several micrometers) detergent-insoluble GJ plaques, where only a proportion of GJs maybe open at a given time^8,9^. When GJs are open, they mediate the exchange of various small signaling molecules, including inositol-3-phosphate (IP3) and cAMP, ions such as Ca^2+^, metabolites, amino acids and microRNAs, between communicating cells. Unlike in HC-mediated auto- or paracrine signaling, GJs allow the signals to directly spread via the cytoplasms of connected cells to synchronize cell functions^1,2^. Many of the same factors that regulate HCs also regulate GJs, but the effects are often opposite. For instance, growth factors, inflammatory cytokines, oxidative stress, ischemia or a moderate elevation of intracellular Ca^2+^ concentration that induces HCs to open may cause closing of GJs^1,4,10^. Thus, processes such as inflammation or wound healing may promote HC-mediated signaling and suppress GJ﻿ communication. In addition to the above channel-dependent functions, the cytoplasmic domain of Cxs can directly interact with other molecules independent of the channel functions, and participate in intracellular signaling cascades that control gene expression, among other functions^11–14^. The biological roles of Cx HCs, GJs and channel-independent functions are still incompletely understood.

Wound healing in skin and mucosa is a critical process that re-establishes the structure and function of the tissue after trauma, and in ideal cases results in fast and complete tissue regeneration. Aberrations of wound healing are common in skin, and include excessive scarring and delayed or deficient wound healing^15,16^. Several animal and human studies have shown that the expression of Cxs is spatiotemporally regulated during wound healing, and that their expression is altered in non-healing chronic wounds and in tissue fibrosis, suggesting that Cxs could play a role in these processes^17–22^. For instance, early downregulation of Cx43 has been linked to proper wound closure^23^ while its upregulation is associated with non-healing chronic wounds^20^. Moreover, suppressing the expression of Cx43 (the most ubiquitous Cx in skin) by antisense oligonucleotides (AS ODN) upon wounding, results in faster skin wound re-epithelialization and closure, and accelerates wound granulation tissue formation^23–25^. Thus, early downregulation of Cx43 expression appears beneficial for wound healing. A wound healing-promoting effect has also been achieved by a mimetic peptide (ACT1) corresponding to the cytoplasmic carboxyl-terminus of Cx43^26–28^. In contrast to Cx43 AS ODN treatment, which reduces the total Cx43 expression resulting in reduced Cx43 GJ and HC abundance^29^, ACT1 peptide specifically interferes with the interaction of Cx43 with a cytoplasmic molecule ZO-1, and may induce HC sequestration while increasing GJs^8,26,27,30,31^. Interestingly, unlike Cx43 AS ODN treatment, ACT1 suppresses collagen deposition, resulting in a reduced fibrotic response *in vivo* ^31^. Thus, Cx43 HCs and GJs may have different effects on the wound healing outcome.

Our recent findings have shown that in human oral mucosal gingival wounds, which heal faster and result in significantly less scarring than skin wounds^32–36^, abundance of Cx43 plaques was strongly suppressed in wound fibroblasts, suggesting that reduced GJ and/or HC function may promote wound healing in gingiva^37^. To further assess the functions of Cx43 in human gingival fibroblasts (GFBLs), we blocked it by mimetic peptides Gap27 or Gap26. These peptides specifically target both Cx43 HCs and GJs at the same time^38^. Interestingly, the peptide treatments strongly modulated the expression of several key genes and proteins associated with wound healing via specific intracellular signaling pathways^37^. Thus, downregulation of Cx43 function may promote the GFBL phenotype conducive for efficient wound healing, but it is not clear whether these functions distinctly depended on Cx43 HCs or GJs. Therefore, the aim of the present study was to characterize Cx43 HCs and GJs in human GFBLs, and determine their roles in regulating fibroblast gene expression relevant for wound healing. We hypothesized that Cx43 HCs and GJs distinctly regulate the expression of wound healing-associated genes in human GFBLs.

## Results

### Immunolocalization of Cx43 GJs and HCs in Human Gingiva *in vivo*

**Figure 1:**
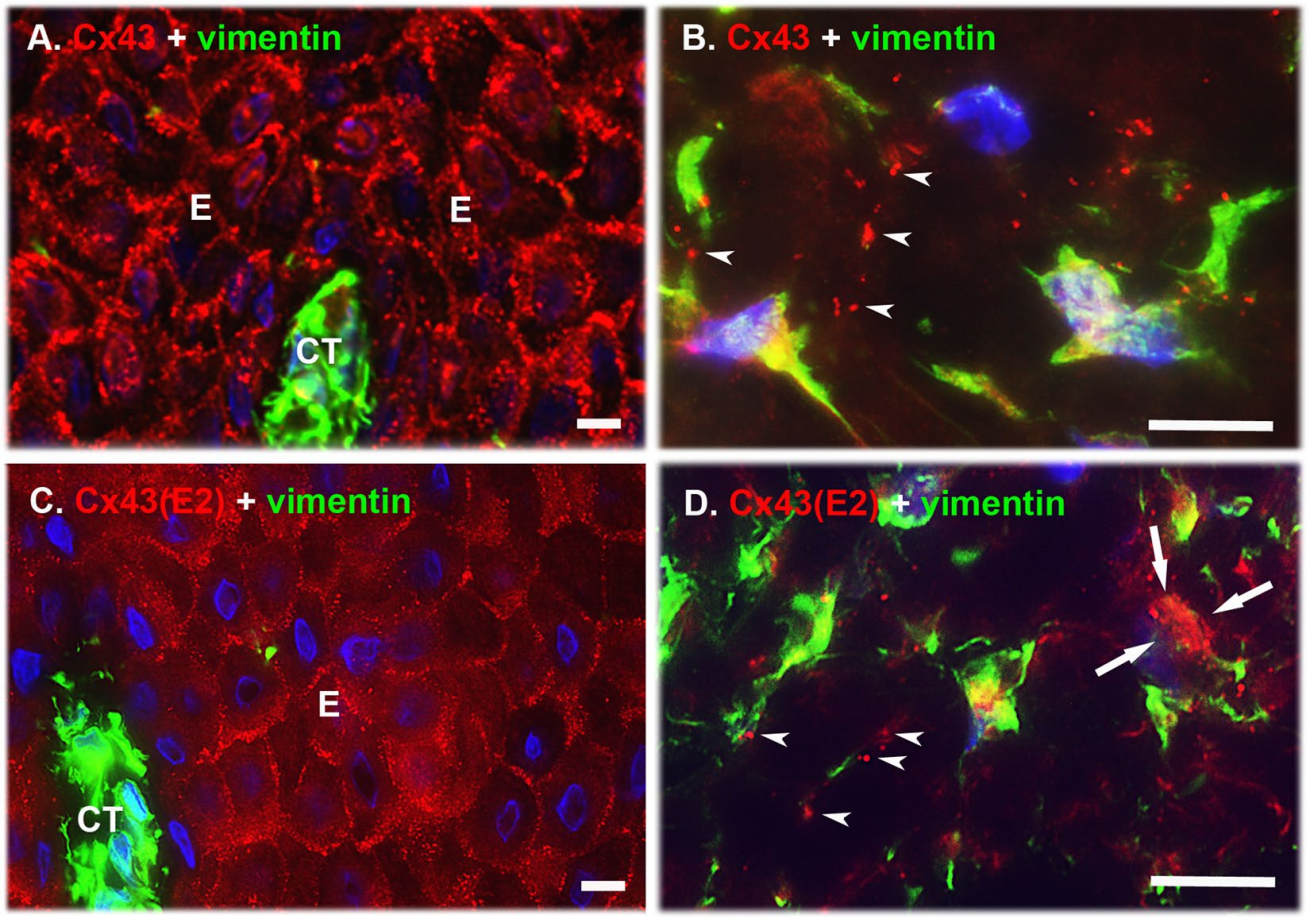
Localization of Cx43 GJs and HCs in human gingiva *in vivo*. Representative images of human gingival tissue sections double immunostained with an antibody recognizing all forms of the Cx43 molecule (Cx43; **A** and **B**) or only HC-associated Cx43 (Cx43(E2); **C** and **D**) and vimentin (a mesenchymal cell marker) in human gingival epithelium (**A** and **C**) and connective tissue (**B** and **D**). In the basal and spinous layers of the epithelium, Cx43 immunostaining localized most abundantly at the cell-cell contacts as fairly large plaque-like structures typical to GJs (**A**). Staining with the Cx43 HC-specific Cx43(E2) antibody also showed localization of Cx43 at the epithelial cell-cell contacts, but the immunopositive structures were markedly smaller. In addition, some punctate immunoreactivity was noted on the cell body of the keratinocytes (**C**). (**B**) In the gingival connective tissue, Cx43 immunoreactivity was also present as large plaque-like staining that mostly localized in the long cellular processes reaching out from the vimentin-positive cells (arrowheads). (**D**) In these cells, Cx43 HCs detected by the Cx43(E2) antibody were also mainly present as plaque-like structures that localized in the cell processes (arrowheads), but some staining was also present on the cell body (arrows). Representative immunostaining images from a minimum of three parallel sections from three individual donors are shown. Nuclear staining (blue) was performed using DAPI. E: Epithelium; CT: Connective tissue. Magnification bars = 10 μm.

We have previously shown that *in vivo* human GFBLs assemble Cx43 into large plaques typical of GJs^37^, but it is unclear whether these cells also possess Cx43 HCs *in vivo*. To this end, we immunostained Cx43 in normal human gingiva using a polyclonal antibody against the cytoplasmic domain of Cx43 that recognizes intracellular, GJ-, and HC-associated Cx43 (total Cx43)^39,40^, or with the Cx43(E2) antibody developed against the E2 extracellular loop that binds only to the HC-associated Cx43^41,42^. Fibroblasts were identified based on their elongated, spindle-shaped morphology, and positive immunoreactivity for vimentin, a molecule highly expressed in fibroblasts^43^. In gingival epithelium, total Cx43 staining localized as large plaques at the cell-cell contact areas of the basal and spinous layers (Fig. 1A). HC-specific Cx43(E2) immunoreactivity was also most abundantly present at the cell-cell contact areas of the basal and spinous layers (Fig. 1C), but the positively stained structures were also markedly smaller than those observed with the antibody recognizing total Cx43 (Fig. 1A). As expected, staining of total Cx43 in connective tissue cells localized to punctate, fairly large plaque-like structures (>1 μm in diameter), typical of GJs, and associated mostly with long cellular processes reaching out from vimentin-positive fibroblast-like cells (Fig. 1B). The cell processes and some areas of the cell body in these cells also showed positive staining with the HC-specific Cx43(E2) antibody (Fig. 1D), but the immunopositive plaque-like structures were in general somewhat smaller (0.5–1 μm in diameter) than those observed with the antibody against total Cx43 (Fig. 1B). Thus, in human gingiva, fibroblasts and keratinocytes assemble Cx43 into large plaques typical of GJs, and to smaller plaques recognized with the Cx43 HC-specific antibody, suggesting the presence of both GJ and HC plaques in human gingival cells *in vivo*.

### Cx43 Assembles into GJs and HCs in Cultured Gingival Fibroblasts

**Figure 2:**
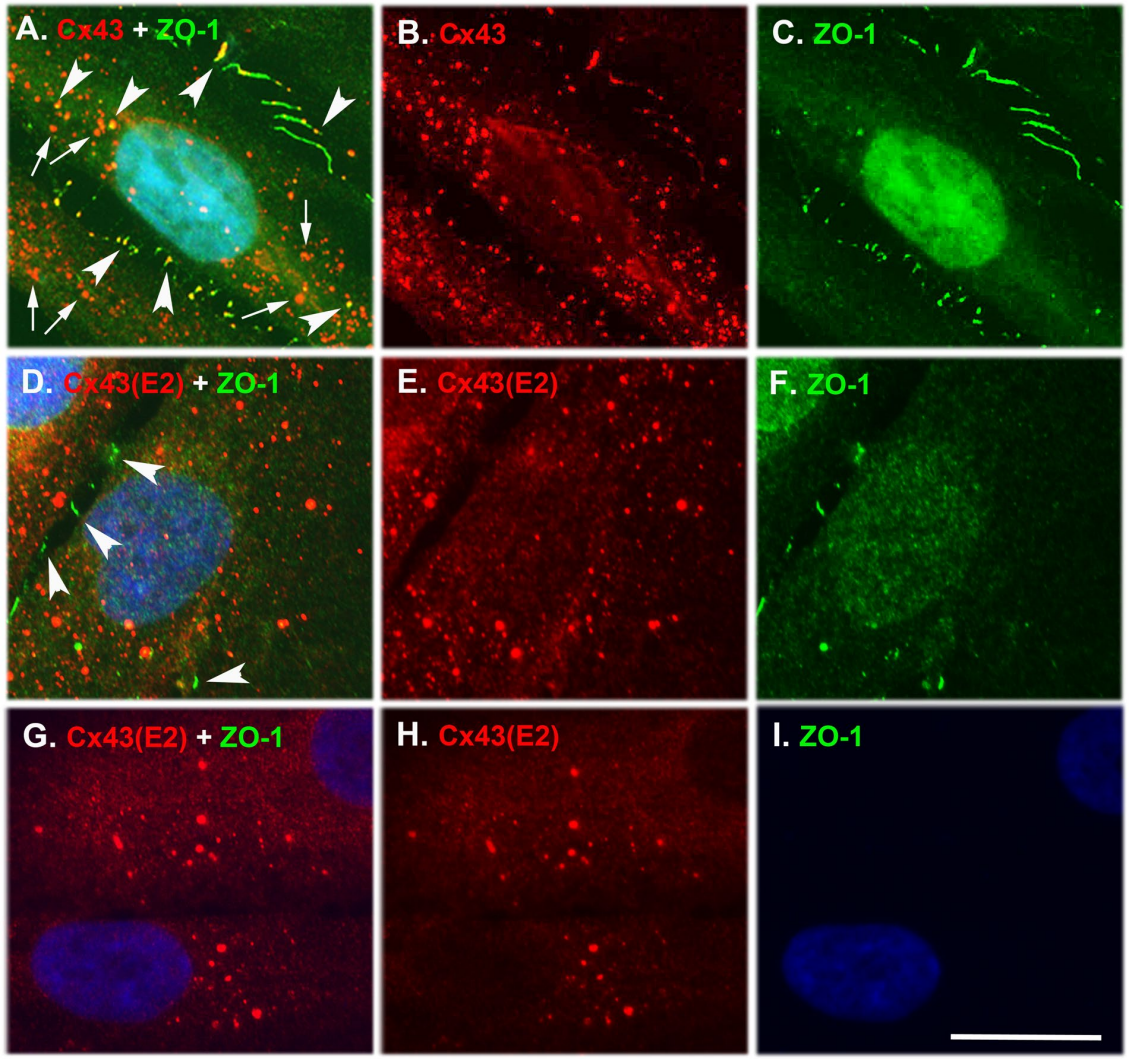
Immunolocalization of Cx43 GJs and HCs in cultured human gingival fibroblasts. (**A**–**C**) Representative images from GFBL-DC cultures fixed and permeabilized with 0.5% Triton X-100 treatment before double immunostaining with antibodies against all forms of Cx43 (red) and ZO-1 (green), indicator of cell-cell contacts. Fibroblasts displayed numerous Cx43-positive plaque-like structures throughout the cell body. Some of these plaques colocalized with ZO-1 staining at apparent cell-cell contacts and over the cell body (arrowheads in A), likely representing GJ plaques. However, Cx43-positive structures that did not colocalize with ZO-1 (arrows) were also present. (**D**–**F**) Representative images of confluent GFBL-DC cultures fixed and permeabilized with 0.5% Triton X-100 treatment before double immunostaining with Cx43(E2) antibody specific for Cx43 HCs (red) and ZO-1 (green). Cx43 HCs were also organized in plaque-like structures present along the cell body, but they were not colocalized with ZO-1 present in the cell-cell contact areas (arrowheads in D). Some cells showed localization of ZO-1 in the nucleus consistent with its function also as a transcription factor. (**G**–**I**) Representative images of a confluent gingival fibroblast (GFBL-DC) culture fixed and then double immunostained with the Cx43(E2) (red) and ZO-1 (green) antibodies without permeabilization with 0.5% Triton X-100 to detect only cell surface-associated Cx43 HCs. In these cells, the Cx43(E2)-positive HC-plaques localized along the cell body, but their number was reduced compared to the permeabilized cells (**D** and **E**). No immunoreactivity for the intracellular ligand ZO-1 was detected as expected (**I**). Nuclear staining (blue) was performed using DAPI. Magnification bar = 10 μm.

We have previously shown that Cx43 is the major Cx and forms functional GJ plaques in confluent monolayer cultures of human GFBLs^37^. In order to assess whether GFBLs also possess Cx43 HCs, we compared the localization of total and HC-associated Cx43 in confluent cultures by immunostaining as above (Fig. 2). To detect cell-cell contacts, we double-immunostained the cells with an antibody against ZO-1, an intracellular molecule involved in the recruitment of Cxs to GJ plaques and an indicator of cell-cell contacts^9^. Results showed that in cells permeabilized with 0.5% Triton X-100 treatment, Cx43-positive structures colocalized with ZO-1 staining between closely positioned cells, and in a few locations on the cell body, likely representing GJ plaques at cell-cell contacts (Fig. 2A–C). In addition, some Cx43 plaques that did not colocalize with ZO-1 were present in the cell body, suggesting that they represented intracellular and/or HC-associated Cx43 (Fig. 2A–C). In order to localize Cx43 HCs in the cell membrane and intracellular pools, or present only on the cell membrane, we permeabilized cells with 0.5% Triton X-100 or left them non-permeabilized, respectively, before immunostaining with the HC-specific Cx43(E2) antibody. In both permeabilized (Fig. 2D–F) and non-permeabilized (Fig. 2G–I) cells, Cx43(E2)-positive plaques were found distributed along the cell body. In permeabilized cells, this staining did not colocalize with ZO-1 (Fig. 2D–F), indicating that Cx43 plaques were non-junctional. As expected, no immunoreactivity for ZO-1, an intracellular molecule, was detected in non-permeabilized cells, confirming that the antibodies did not have access to the cytosol in non-permeabilized cells (Fig. 2G and I). Thus, cultured human GFBLs possess both Cx43 GJ and HC plaques that associate with cell-cell contacts and non-junctional cell membranes, respectively.

To further characterize Cx43 GJs and HCs, we cultured GFBLs in high density (HD; 100% confluence) to allow cells to form abundant GJ-mediated cell-cell contacts, or in low density (LD; 10% confluence), which results in the formation of fewer cell-cell contacts and GJs. To further study the localization of Cx43 into GJs that are typically present in cell membrane lipid rafts, a set of cultures was pretreated with 1% Triton X-100 to remove non-lipid raft-associated Cx43 (HCs and cytoplasmic pool)^44–48^. Results showed that Cx43 assembled into GJs and HCs in both HD and LD cultures (Supplementary Fig. S1A-J), and that cell density did not affect Cx43 expression at mRNA (Supplementary Fig. S1K) or protein (Supplementary Fig. S1L and M) levels. However, immunostaining and Western blotting confirmed that increasing the cell density caused a redistribution of Cx43 from a mostly non-junctional (representing intracellular and HC pool of Cx43) to a mostly junctional pool (representing GJ fraction of Cx43) (Supplementary Fig. S1A-J and L-N) as expected.

### Gingival Fibroblasts Possess Functional Cx43 GJs and HCs

**Figure 3:**
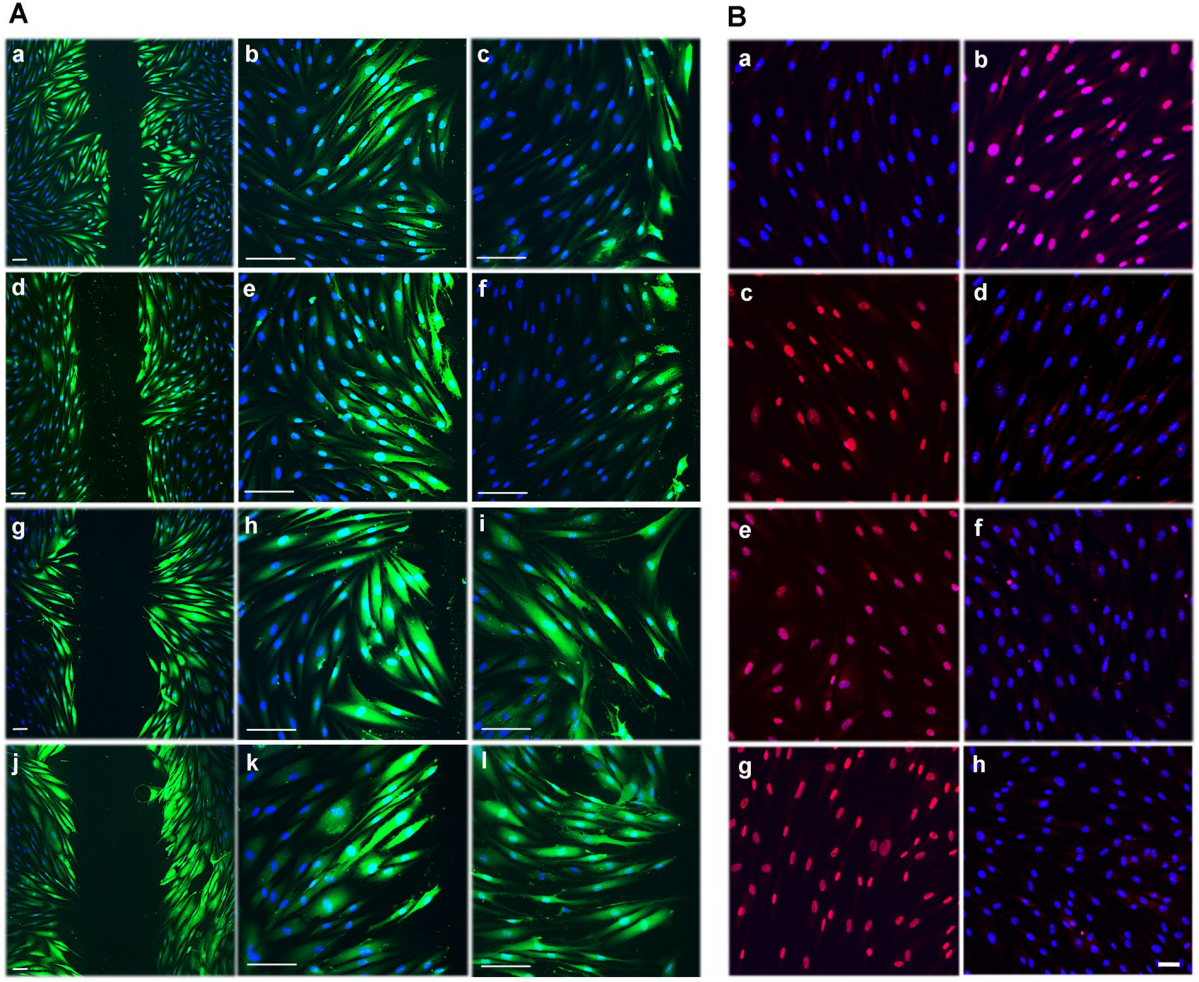
Gingival fibroblasts have functional Cx43 GJs and HCs. (**A**) Confluent GFBL-DC cultures maintained in DMEM were scrape-loaded with Lucifer Yellow (green) in the presence of vehicle (dH_2_O) (a and b), a non-specific Cx inhibitor meclofenamic acid (MFA; 50 μM) (c), Gap27 control peptide (150 μM) (d and e), Cx43 mimetic peptide Gap27 (150 μM) (f), TAT-Gap19 control peptide (400 μM) (g and h), TAT-Gap19 (400 μM) (i), non-immune rabbit IgG (1 mg/mL) (j and k), or Cx43(E2) antibody (1 mg/mL) (l), and dye transfer via GJs was followed for 5 min. Treatment of cells with MFA (c) or Gap27 (f) markedly reduced dye transfer as compared to corresponding control samples (a and b or d and e, respectively), while TAT-Gap19 (i) or Cx43(E2) (l) had no effect, as expected. (**B**) Confluent GFBL-DC cultures incubated in DMEM (containing 1.8 mM Ca^+2^) (a) or low Ca^+2^-containing medium (EMEM supplemented with 180 nM Ca^+2^) (b) in the presence of Cx HC-permeable Propidium Iodide (PI; 2.5 mM, red) for 20 min. No dye uptake was noted in cells incubated in DMEM (a), while incubation of cells in EMEM potently induced dye uptake (b). (c-h) Fibroblasts were incubated in EMEM and treated with Gap27 control peptide (c) or Gap27 (150 μM) (d), TAT-Gap19 control peptide (e) or TAT-Gap19 (400 μM) (f), and non-immune rabbit IgG (g) or Cx43(E2) antibody (1 mg/mL) against Cx43 HCs (h). Gap27 (d), TAT-Gap19 (f), and Cx43(E2) antibody (h) potently blocked Cx HC-mediated dye uptake as expected. Results show representative images from a minimum of three repeated experiments. For the experiments, cells were pretreated with the inhibitors or controls for 1 h before the experiments. Nuclear staining (blue) was performed using DAPI. Magnification bars in A = 30 μm (a, d, g and j) and 50 μm (b, c, e, f, h, i, k and l); in B = 20 μm.

Having established that GFBLs possess both Cx43 GJs and HCs, we wanted to reveal their functionality. To test Cx43 GJs, cells were scrape-loaded with Lucifer Yellow and dye transfer was assessed by fluorescence microscopy^49^. After 5 min, GFBLs in control samples showed avid Lucifer Yellow transfer extending to several cells from the wound edge (Fig. 3A; a, b, d, e, g, h, j, k). However, when cells were pretreated with MFA (Fig. 3A; c), a non-specific Cx inhibitor^50^, or Gap27 (Fig. 3A; f) that specifically binds to the Cx43 extracellular loop and blocks its GJ and HC functions^38,51,52^, dye transfer was potently blocked. As expected, dye transfer was unaffected when cells were treated with TAT-Gap19 peptide (Fig. 3A; i) or with the Cx43(E2) antibody (Fig. 3A; l), which both specifically block Cx43 HC functions without affecting GJs^41,42,53,54^. Thus, human GFBLs possess functional Cx43 GJs that can be blocked with Gap27 or MFA, while the HC-targeting TAT-Gap19 and Cx43(E2) antibody have no effect.

To assess Cx43 HC functions, GFBLs were incubated in low Ca^2+^ medium (180 nM Ca^2+^) to induce the opening of Cx HCs, and then treated with HC-permeable Propidium Iodide (PI)0^49^. Dye transfer via HCs was assessed after 20 min using fluorescence microscopy. While cells kept in high Ca^2+^-containing medium (1.8 mM Ca^2+^) did not show any dye transfer as expected (Fig. 3B; a), practically all GFBLs in low Ca^2+^ displayed nuclear PI staining (Fig. 3B; b), indicating efficient HC-mediated dye transfer. To assess the involvement of Cx43 HCs in this process, we then treated cells with PI in the presence of Gap27 (Fig. 3B; d), TAT-Gap19 (Fig. 3B; f), or Cx43(E2) antibody (Fig. 3B; h). All treatments completely blocked dye transfer, indicating that it occurred via Cx43 HCs. Corresponding control treatments did not have any effect on the dye transfer (Fig. 3B; c, e, g). Thus, GFBLs also have functional Cx43 HCs that can be blocked with Gap27, which also blocks Cx43 GJs, and TAT-Gap19 and Cx43(E2) antibody, that do not affect Cx43 GJs in these cells.

### Targeting of Cx43 with TAT-Gap19 Significantly Regulates Gene Expression Similar to Gap27 in Gingival Fibroblasts

**Figure 4:**
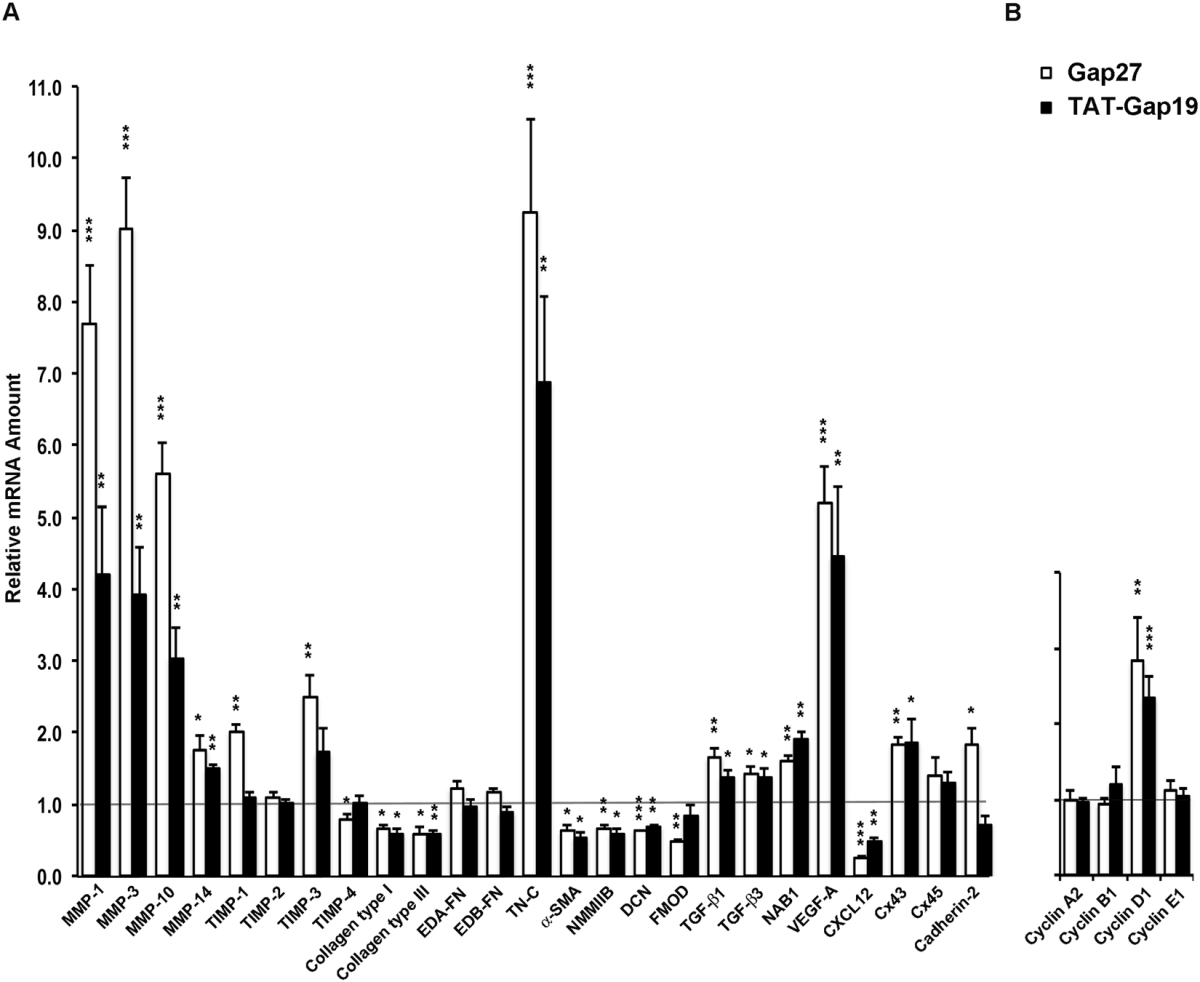
Gap27 and TAT-Gap19 induce partially similar gene expression response in human gingival fibroblasts. Confluent GFBL-DC cultures were treated with Gap27 or control peptide (150 μM), and TAT-Gap19 or control peptide (400 μM) for 24 h, and the expression of a set of genes involved in (**A**) wound healing and (**B**) regulation of cell cycle was analyzed by qPCR. Results represent mean +/− s.e.m. of amount of mRNA relative to control peptide-treated cells from a minimum of three repeated experiments. Statistical testing was performed by comparison of Gap27- or TAT-Gap19-induced gene expression change relative to the controls (*p < 0.05, **p < 0.01, ***p < 0.001; two-tailed *t*-test). Horizontal line indicates relative amount of mRNA for the control peptide-treated samples. EDA-FN: Extra Domain A-Fibronectin; EDB-FN: Extra Domain B-Fibronectin; TN-C: Tenascin-C; α-SMA: α-Smooth Muscle Actin; NMMIIB: Non-Muscle Myosin IIB; DCN: Decorin; FMOD: Fibromodulin; NAB1: NGFI-A Binding Protein-1; VEGF-A: Vascular Endothelial Growth Factor-A.

We have previously shown that Gap27 treatment significantly regulates mRNA and protein expression of a set of key wound healing-associated genes in human GFBLs cultured in HD^37^. However, it is not known whether the gene expression change is mediated by Cx43 GJs or HCs. Therefore, we treated confluent GFBL cultures with Gap27 (150 μM) to block both Cx43 HCs and GJs, or TAT-Gap19 (400 μM), which blocks its HC function, and analyzed gene expression by qPCR. Results showed that Gap27 treatment significantly changed the expression of 21 of the 25 genes analyzed, while having no effect on four of the assessed genes (TIMP-2, EDA-FN, EDB-FN, and Cx45) (Fig. 4A), which is consistent with our previous data^37^. Similar to Gap27, TAT-Gap19 treatment induced significant up or downregulation of 16 (MMP-1, -3, -10, -14, Collagen type I, Collagen type III, Tenascin-C, Decorin, α-SMA, NMMIIB, TGF-β1, TGF-β3, NAB1, VEGF-A, CXCL12, and Cx43) of the 21 Gap27-responsive genes, although the magnitude of change relative to the untreated cells slightly varied between Gap27- and TAT-Gap19-treated cells (Fig. 4A). Findings from set of experiments showed that TAT-Gap19 responses were concentration-dependent from 200 µM up to 500 µM (Supplementary Fig. S2A). Similarly, Gap19 peptide that was not linked with the TAT cell-penetrating peptide caused concentration-dependent gene expression changes, albeit at a slightly lower efficiency (Supplementary Fig. S2B). Thus, the expression of the above 16 genes maybe regulated via blocking of the Cx43 HC function by Gap27 and TAT-Gap19. In contrast, the expression of the five genes (TIMP-1, -3, -4, Cadherin-2, and Fibromodulin), which were significantly regulated by Gap27, but not by TAT-Gap19, may depend on Gap27-mediated inhibition of GJ functions. The expression of the four genes (TIMP-2, EDA-FN, EDB-FN, and Cx45) not regulated by Gap27 was also unaffected by TAT-Gap19 (Fig. 4A), confirming that their expression is not regulated by Cx43 GJs or HCs in these cells.

### Modulation of Cell Cycle by Gap27 and TAT-Gap19 in Gingival Fibroblasts

To assess whether gene expression changes caused by blocking of Cx43 function by the mimetic peptides were associated with cell cycle modulation, we performed qPCR for Cyclin A2, Cyclin B1, Cyclin D1, and Cyclin E1, four genes that are distinctly expressed during different stages of cell cycle^55^. To this end, we treated confluent GFBL cultures with Gap27 (150 μM) or TAT-Gap19 (400 μM), and analyzed gene expression by qPCR. Results showed that Cyclin D1, a gene that regulates transition from G1 to S phase of the cell cycle^55^, was significantly elevated by both Gap27 and TAT-Gap19 while the other cyclins did not show significant change compared to control samples (Fig. 4B).

### Targeting of Cx43 with TAT-Gap19 Modulates ERK Signaling Pathway Similar to Gap27 in Gingival Fibroblasts

**Figure 5:**
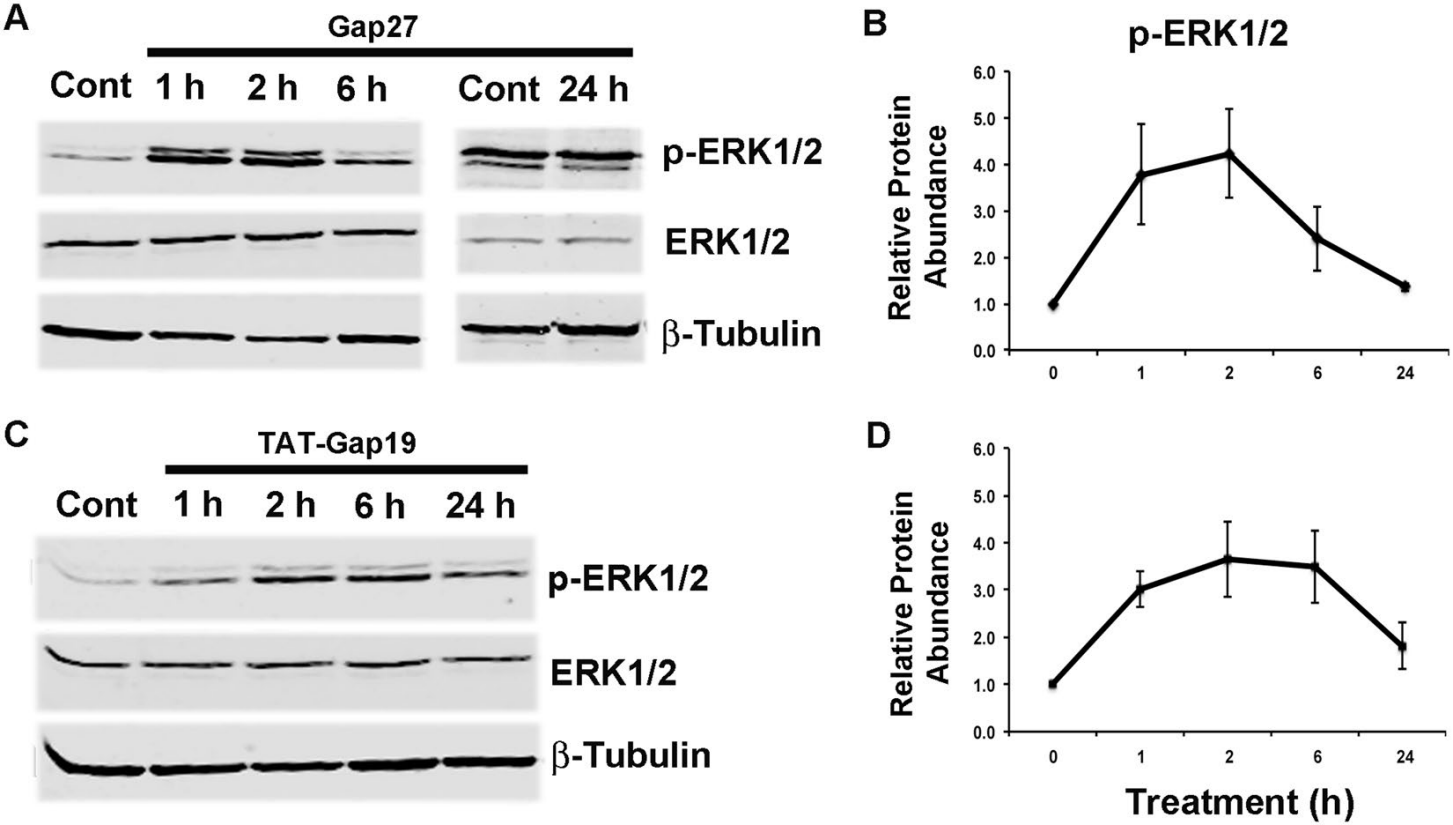
Western blotting analysis of activation of ERK1/2 of the MAPK pathway by Cx43 GJs and HCs in gingival fibroblasts. Confluent GFBL-DC cultures were treated with Gap27 or control peptide (150 μM) (**A**), and TAT-Gap19 or control peptide (400 μM) (**C**) for 1, 2, 6, and 24 h. Gap27 treatment was used as a positive control for signaling pathway activation. Cell lysates were analyzed for protein levels of total ERK1/2 and phosphorylated ERK1/2 (p-ERK1/2). Cropped blots from different parts of the same or different blots are separated by white spaces. Full-length blots are displayed in Supplementary Figure S5. (**B** and **D**) Quantitation of p-ERK1/2 abundance relative to its total levels at time 0 (control samples) and at 1, 2, 6, and 24 h after treatment. Sample loading was normalized for β-Tubulin levels. Results represent mean +/− s.e.m. of relative protein abundance from two repeated experiments.

We have previously shown that most gene expression changes induced by Gap27 treatment are linked to the activation of the ERK1/2 signaling pathway in GFBLs^37^. Therefore, having established that targeting Cx43 HCs with TAT-Gap19 regulates the expression of a set of wound healing-associated genes similar to Gap27, we wanted to find out whether TAT-Gap19 also activates the ERK1/2 signaling pathway. Similar to Gap27, TAT-Gap19 treatment already markedly induced phosphorylation of ERK1/2 after 1 h of treatment. With both treatments, ERK1/2 activation lasted for at least 6 h, before returning to the level of untreated cells by 24 h (Fig. 5). Thus, in GFBLs, targeting Cx43 with TAT-Gap19 or Gap27 treatment similarly involves activation of the ERK1/2 signaling pathway.

### Distinct Involvement of ERK1/2 Signaling Pathway in Modulation of Cx43-Regulated Genes in Gingival Fibroblasts

**Figure 6:**
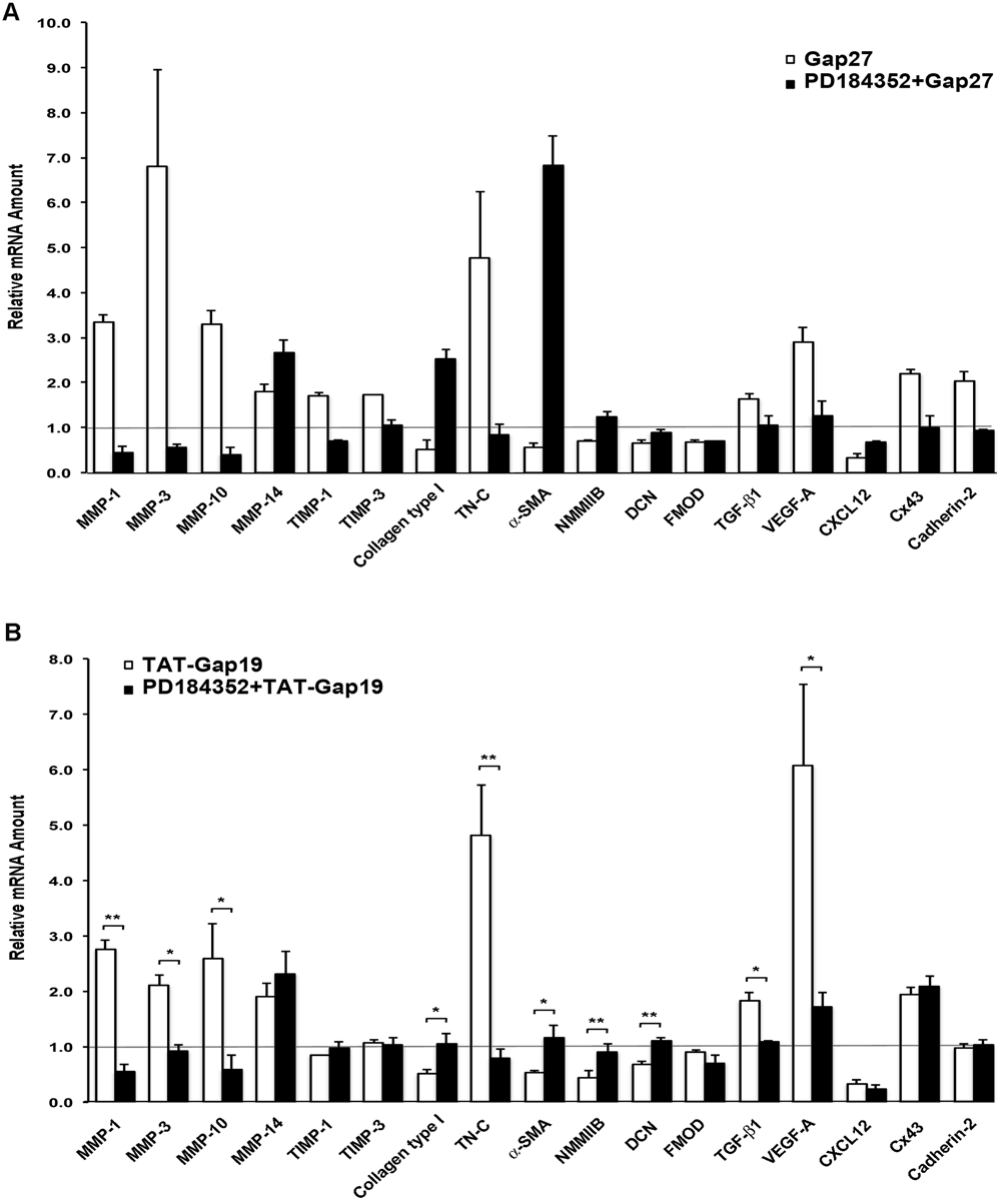
Modulation of Gap27 and TAT-Gap19-regulated gene expression by pharmacological inhibitor of ERK1/2 signaling pathway. (**A** and **B**) Confluent cultures of GFBL-DC were treated with Gap27 (150 μM) (**A**), or with TAT-Gap19 (400 μM) (**B**) with or without MEK1/2 inhibitor (PD184352; 10 μM) for 24 h, and amount of mRNA for wound healing-associated genes was analyzed by qPCR. Results represent mean +/− s.e.m. of relative amount of mRNA from two repeated experiments in (**A**) and three repeated experiments in (**B**) (*p < 0.05, **p < 0.01; two-tailed *t*-test). Horizontal line indicates relative mRNA expression for the control samples (vehicle-treated cells; 0.1% DMSO). TN-C: Tenascin-C; α-SMA: α-Smooth Muscle Actin; NMMIIB: Non-Muscle Myosin IIB; DCN: Decorin; FMOD: Fibromodulin; VEGF-A: Vascular Endothelial Growth Factor-A.

In order to further study the role of the ERK1/2 pathway in Gap27 and TAT-Gap19-induced gene expression, we blocked the ERK1/2 pathway by PD184352 in Gap27 or TAT-Gap19-treated cells and assessed gene expression changes by qPCR. We specifically assessed the expression of a set of 17 wound healing-related genes that were previously significantly modulated (with a minimum 1.5-fold change threshold) by Gap27 treatment^37^. These include 13 genes commonly regulated by Gap27 and TAT-Gap19. As previously reported^37^, inhibition of the ERK1/2 pathway by PD184352 blocked Gap27-induced expression changes of 13 of these genes (MMP-1, -3, -10, TIMP-1, -3, Collagen type I, Tenascin-C, α-SMA, NMMIIB, TGF-β1, VEGF-A, Cx43, and Cadherin-2), while the expression of four genes (MMP-14, Decorin, Fibromodulin, and CXCL12) was not regulated by this pathway (Fig. 6A). Likewise, PD184352 treatment significantly blocked TAT-Gap19-induced expression changes in nine of the above 13 genes (MMP-1, -3, -10, Collagen type I, Tenascin-C, α-SMA, NMMIIB, TGF-β1, and VEGF-A). While Gap27 and TAT-Gap19 bind to different domains in the Cx43 molecule, their common property is their ability to block function of Cx43 HCs^53^. Therefore, expression of the above nine genes maybe regulated by Gap27 and TAT-Gap19-induced suppression of Cx43 HC function and subsequent activation of the ERK1/2 pathway. In contrast, expression changes of three genes (MMP-14, Decorin, and CXCL12) commonly regulated by Gap27 and TAT-Gap19 were not affected by PD184352 treatment, suggesting the involvement of an ERK1/2-independent Cx43 HC-regulated pathway (Fig. 6B). Both Gap27 and TAT-Gap19 treatments significantly induced expression of Cx43. Interestingly, however, the Gap27-induced Cx43 mRNA increase was sensitive to PD184352 treatment while TAT-Gap19 was not, suggesting that Gap27 and TAT-Gap19 induce Cx43 expression by distinct mechanisms in GFBLs.

### Inhibition of ATP Signaling Partially Recapitulates Gene Expression Changes Induced by TAT-Gap19 in Gingival Fibroblasts

**Figure 7:**
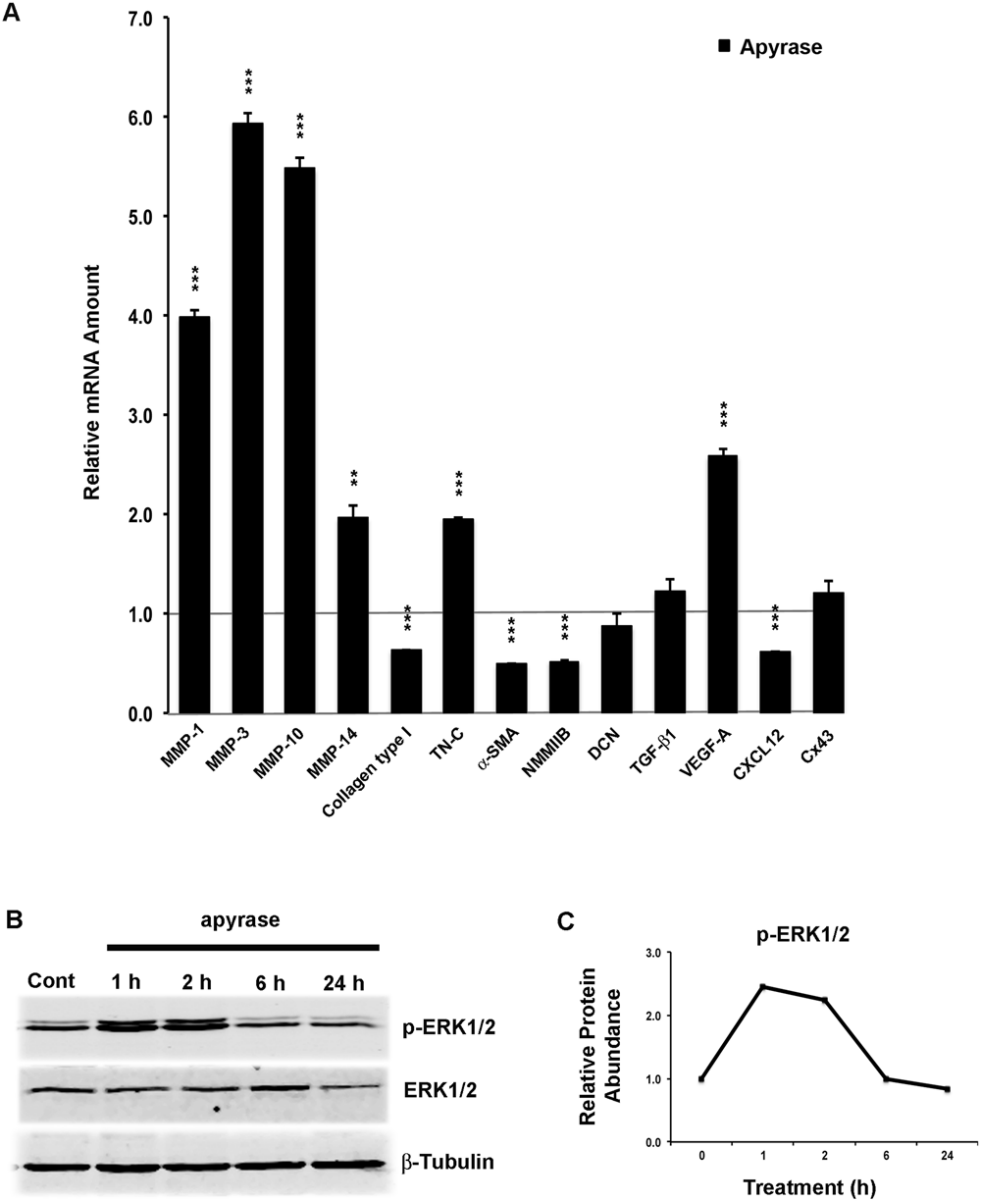
Blocking of ATP signaling pathway significantly modulates amount of mRNA for key wound healing-associated genes and activates the ERK1/2 pathway. (**A**) Results show qPCR analysis of relative amount of mRNA in confluent GFBL-DC cultures treated with apyrase (1 U/mL) relative to vehicle control (dH_2_O) for 24 h. Results represent mean +/− s.e.m. from a minimum of three repeated experiments (**p < 0.01, ***p < 0.001; two-tailed *t*-test). EDA-FN: Extra Domain A-Fibronectin; EDB-FN: Extra Domain B-Fibronectin; TN-C: Tenascin-C; α-SMA: α-Smooth Muscle Actin; NMMIIB: Non-Muscle Myosin IIB; DCN: Decorin; FMOD: Fibromodulin; NAB1: NGFI-A Binding Protein-1; VEGF-A: Vascular Endothelial Growth Factor-A. (**B**) Results show Western blotting analysis of ERK1/2 pathway activation in confluent GFBL-DC cultures that were treated with apyrase (1 U/mL) or vehicle control (dH_2_O) for 1, 2, 6, and 24 h. Cell lysates were analyzed for protein levels of total ERK1/2 and phosphorylated ERK1/2 (p-ERK1/2). (**C**) Quantitation of p-ERK1/2 abundance relative to its total levels at time 0 (control sample) and at 1, 2, 6, and 24 h after treatment. Sample loading was normalized for β-Tubulin levels. Cropped blots from different parts of the same or different blots are separated by white spaces. Full-length blots are displayed in Supplementary Figure S5.

Cx43 HCs are known to mediate the release from cells of ATP, which is a powerful auto- and paracrine signaling molecule. Gap27 and TAT-Gap19 have been shown to efficiently block the Cx43 HC-mediated ATP release in various cells^53,56^. Furthermore, ATP-regulated cell signaling has been linked to the ERK1/2 pathway^57,58^. Therefore, to study whether suppression of ATP-mediated signaling is important in the regulation of gene expression by Gap27 and TAT-Gap19, we treated cells with apyrase that selectively degrades extracellular ATP^59,60^, and assessed gene expression changes relative to vehicle-treated cells by qPCR. The analysis focused on the above 13 genes commonly regulated by TAT-Gap19 and Gap27. Results showed that 10 of these genes (MMP-1, -3, -10, -14, Collagen type I, Tenascin-C, α-SMA, NMMIIB, VEGF-A, and CXCL12) were also significantly up or downregulated by apyrase (Fig. 7A), which was similar to Gap27 and TAT-Gap19 treatment. In contrast, when cells were pretreated with TAT-Gap19, apyrase treatment did not have any additional effect on the expression of these genes (Supplementary Fig. S3). This suggests that the expression of the above 10 genes is regulated by the blocking of HC-mediated ATP release by Gap27 and TAT-Gap19. In contrast, TAT-Gap19 and Gap27-induced expression of Decorin, TGF-β1, and Cx43 was not affected by apyrase treatment, suggesting the involvement of other mechanisms.

### Modulation of mRNA Abundance of ATP and Adenosine Receptors by Gap27 and TAT-Gap19 in Gingival Fibroblasts

In order to find out whether Gap27 and TAT-Gap19 treatments also affect the expression of cell surface receptors involved in ATP or adenosine (a metabolite of ATP) signaling^61,62^, we performed qPCR for the Cx43-mimetic peptide-treated samples as above. The peptide treatments did not significantly modulate abundance of mRNA of ATP receptors P2X4 and P2X7 or adenosine receptor P1A2bR and adenosine deaminase (ADA), a modulator of adenosine signaling, compared to control samples (Supplementary Fig. S4). However, unlike TAT-Gap19, Gap27 treatment caused a significant down- and upregulation of CD39 and CD73 mRNA abundance, respectively, two receptors involved in generation of adenosine from ATP^61,62^, compared to control samples (Supplementary Fig. S4). Abundance of mRNA of ATP receptors P2Y1 and P2Y2 and adenosine receptors P1A1R, P1A2aR, and P1A3R in GFBLs was negligible and was not explored further.

### Blocking of ATP Signaling Activates ERK1/2 Signaling Pathway Similar to Cx43 Mimetic Peptides in Gingival Fibroblasts

The findings from above showed that blocking of Cx43 HC function with TAT-Gap19 or Gap27 treatment causes activation of the ERK1/2 signaling pathway (Fig. 5), and blocking ATP signaling with apyrase results in a similar gene expression response (10 out of 13 genes) as treatment with Gap27 or TAT-Gap19 (Fig. 7A). Therefore, we wanted to ask whether the apyrase-modulated gene expression response was also associated with activation of the ERK1/2 signaling pathway. To this end, we treated GFBLs with apyrase and assessed ERK1/2 phosphorylation over time by Western blotting. Similar to Gap27 and TAT-Gap19 (Fig. 5), apyrase treatment already markedly induced phosphorylation of ERK1/2 after 1 h of treatment, which lasted for at least 6 h, before returning to the level of untreated cells by 24 h (Fig. 7B,C).

Collectively, the findings indicate that in GFBLs, the expression of a set of wound healing-related genes (MMP-1, -3, -10, -14, Collagen type I, Tenascin-C, α-SMA, NMMIIB, VEGF-A, CXCL12, TGF-β1, and Decorin) is regulated by Cx43 HCs (genes that were responsive to both Gap27 and TAT-Gap19). The expression change of the majority of these genes (except for TGF-β1 and Decorin) was ATP-dependent (ATP inhibition by apyrase caused a similar expression change as blocking of Cx43 HC function). Among Cx43 HC-regulated genes that were dependent on inhibition of ATP activity, the expression of eight out of 10 genes (MMP-1, -3, -10, Collagen type I, Tenascin-C, α-SMA, NMMIIB, and VEGF-A) was also regulated by the ERK1/2 pathway (Table 1). Our findings also suggest that the expression of four genes (TIMP-1, -3, Cadherin-2, and Fibromodulin) is regulated by Cx43 GJs (genes that were only responsive to Gap27 but not to TAT-Gap19). Out of these genes, the expression change of TIMP-1, -3, and Cadherin-2 was ERK1/2 mediated. Among the studied genes, the expression of four genes (TIMP-2, EDA-FN, EDB-FN, and Cx45) was not regulated by Cx43 GJs or HCs (Table 1). The regulation of Cx43 expression in GFBLs by Cx mimetic peptides appears complex. Both Gap27 and TAT-Gap19 significantly upregulate Cx43 expression, but the Gap27-mediated response was ERK-dependent while the response to TAT-Gap19 was not. Furthermore, Cx43 expression was not affected by apyrase treatment (ATP signaling). The gene expression changes caused by Gap27 and TAT-Gap19 were commonly associated with significantly increased expression of Cyclin D1, a modulator of cell cycle^55^. In addition, Gap27, but not TAT-Gap19, also modulated the expression of CD39 and CD73, two receptors involved in ATP metabolism to adenosine^61,62^.

**Table 1:** Summary of Involvement of ATP and ERK1/2 Signaling Pathways in Cx43 HC- or GJ-Regulated Genes.

**Cx43-independent Genes**	**Cx43 GJ-regulated Genes**		**Cx43 HC-regulated Genes**			
TIMP-2 EDA-FN EDB-FN Cx45	**ERK1/2-mediated**	**Not ERK1/2- mediated**	**ATP-dependent**		**Not ATP-dependent**	
	TIMP-1 TIMP-3 Cadherin-2	FMOD	**ERK1/2- mediated**	**Not ERK1/2- mediated**	**ERK1/2- mediated**	**Not ERK1/2- mediated**
			MMP-1	MMP-14	TGFβ−1	DCN
			MMP-3	CXCL12		
			MMP-10			
			Collagen- type I			
			TN-C			
			α-SMA			
			NMMIIB			
			VEGF-A			

Table shows a summary of expression changes of key wound healing-associated genes whose regulation was mediated by Cx43 GJs (genes that were only responsive to Gap27 but not to TAT-Gap19) or by Cx43 HCs (genes that were responsive to both Gap27 and TAT-Gap19 treatments) in human GFBLs. These genes are further categorized based on whether or not their expression change was inhibited by apyrase (ATP-regulated genes) or ERK1/2 inhibitor (ERK1/2-mediated). Results were obtained from qPCR analysis of relative amount of mRNA in confluent GFBL-DC cultures treated with Gap27 (150 μM) or TAT-Gap19 (400 μM) with or without MEK1/2 signaling pathway inhibitor (PD184352; 10 μM), and with ATP inhibitor (apyrase; 1 U/mL) for 24 h, and show results relative to control peptide/vehicle treated samples. EDA-FN: Extra Domain A-Fibronectin; EDB-FN: Extra Domain B-Fibronectin; FMOD: Fibromodulin; TN-C: Tenascin-C; α-SMA: α-Smooth Muscle Actin; NMMIIB: Non-Muscle Myosin IIB; VEGF-A: Vascular Endothelial Growth Factor-A; DCN: Decorin.

## Discussion

The findings in the present study showed that Cx43 assembles into distinct plaques in GFBLs *in vivo* and *in vitro*. Based on immunostaining of tissue sections and GFBL cultures using Cx43 antibodies that recognizes total Cx43 or only HC-associated Cx43, we further showed that some of these plaques were composed of Cx43 HCs while others localized to cell-cell contacts likely representing Cx43 GJ plaques. It is widely accepted that Cx43 forms GJ plaques *in vivo* and *in vitro* in various cells and tissues^9^. Evidence from atomic force microscopy has also suggested the presence of up to 2 μm^2^ HC plaques in cardiac cells *in vivo* ^63^, but it remains unclear whether such HC plaques also exist in other tissues or cells. Elegant human and animal epithelial and fibroblast cell culture studies have shown that to form GJs, Cxs are first transported to the cell membrane as HCs, where they then cluster into plaques and assemble into GJs^12,64^. These Cx plaques can be several micrometers in size and, therefore, unlike individual Cxs, are detectable by immunostaining^9^. Thus, some of the Cx43 plaques noted in GFBLs are likely composed of both Cx HCs and GJs. However, in cultured GFBLs, the localization of Cx43 HC plaques was mainly noted along the cell body not associated with cell contacts, suggesting that these plaques are composed of HCs only. Cxs have not been shown to organize into plaque-like structures intracellularly^12^, suggesting that the noted plaques are associated with the cell membrane. This is further supported by our findings also showing Cx43 HC staining in cells not permeabilized by Triton X-100 prior to staining. In any case, our biochemical fractionation and Cx43 immunoblotting data (Supplementary Fig. S1) indicate the presence of two distinct pools of Cx43 in GFBLs, as described previously for other cell types^39^. These pools likely represent GJs that can be mostly found in the Triton X-100 insoluble cell membrane lipid raft fraction, while the detergent-soluble non-lipid raft pool consists of Cx43 HCs present on the membrane and intracellularly^45^. Thus, collectively, the above findings show for the first time the presence of both Cx43 GJs and HCs in GFBLs *in vivo* and *in vitro*.

Our GJ and HC-specific dye transfer experiments and functional blocking of Cx43 GJs and HCs by specific peptides further indicate that Cx43 GJs and HCs are functional in GFBLs. Interestingly, however, in cultures where GFBLs possess both Cx43 GJs and HCs abundantly, specific blocking of both Cx43 GJ and HC function by Gap27 or only HC function by TAT-Gap19 showed in general a similar effect on gene expression, and this was mediated by the activation of the ERK1/2 pathway. Thus, the Cx43 HC-mediated pathway appears to play an important role in the regulation of wound healing-associated genes in cultured GFBLs. Cx43 HCs are important conduits for auto- and paracrine signaling mediated by ATP^65^. Our data also suggests that the gene expression response and activation of the ERK1/2 pathway by Cx43 HC-blocking peptides depends on reduced ATP signaling by GFBLs. This is based on the findings that inhibition of ATP activity by apyrase caused in general a similar gene expression response and activation of the ERK1/2 pathway as the blocking of Cx43 HCs by the peptides. Furthermore, inhibition of ATP signaling by apyrase did not have an additional effect over the gene expression change induced by Cx43 HC blocking by TAT-Gap19 (Supplementary Fig. S3). Thus, in cultures of human GFBLs where the cells possess functional Cx43 GJs and HCs, the blocking of Cx43 HC function and ATP signaling by Cx43 mimetic peptides causes a robust ERK1/2-dependent change in the expression of a set of wound healing-associated genes. These changes include significant upregulation of MMPs (MMP-1, -3, and -10) that modulate inflammation and tissue remodeling^66,67^, TN-C that regulates cell migration and suppresses fibrosis^68^, and VEGF-A that promotes angiogenesis^69^, and downregulation of Collagen type I, α-SMA and NMMIIB that are associated with fibrosis^70^. Thus, the inhibition of Cx43 HC-dependent ATP signaling by Cx43 HC-specific blocking peptides may be used to promote a gene expression response that may be beneficial for fast and scarless wound healing. The targeting of Cx43 HCs to promote wound healing by blocking peptides may also have beneficial effects via other mechanisms. ATP released by connective tissue and inflammatory cells is an important pro-inflammatory signal during the early stages of wound healing^71^. In certain animal models, the blocking of Cx43 function prevents this pro-inflammatory ATP release^72^. This is significant as increased inflammation delays wound healing and promotes fibrosis and excessive scar formation^73^. In line with the above, the transient blocking of Cx43 functions has been shown to promote experimental wound healing and reduce fibrosis^26,31,74^. Interestingly, however, fibroblasts from two fibrotic skin conditions (hypertrophic scars and keloids) display reduced Cx43 levels and GJ-mediated intercellular communication, suggesting that the normal GJ functions of Cx43 may be important for normal fibroblast function^75^. Therefore, our finding that specifically targeting Cx43 HC function without affecting GJs promotes the expression of wound healing-related genes in fibroblasts may provide a novel specific target to modulate functions beneficial for wound healing. Specific targeting of Cx43 HCs may also be desirable as the blocking of Cx43 GJ functions may have more systemic side effects, including impaired cardiac function^76,77^.

Interestingly, a subset of genes in GFBLs (TIMP-1, TIMP-3, Cadherin-2 and Fibromodulin) was significantly modulated by Gap27, but not by TAT-Gap19, suggesting that their expression is regulated by Cx43 GJs. This was not associated with differential cell cycle regulation by the two peptides, as both caused a significant increase in Cyclin D1 mRNA abundance. However, Gap27 treatment was distinctly associated with a significant change in mRNA abundance of CD39 and CD73, two receptors that metabolize ATP to adenosine^61,62^. Importance of this pathway in Cx43-regulated gene expression warrants further investigation.

While our results showed that Cx43 expression was significantly upregulated with both Gap27 and TAT-Gap19 treatments, the former response was ERK-dependent but the latter was not. Furthermore, Cx43 expression was not affected by apyrase-mediated extracellular ATP degradation that also induced ERK activation, a situation mimicking inhibition of HC-mediated ATP release from cells. Therefore, the regulation of Cx43 expression may involve distinct Cx43 GJ and HC-mediated feedback mechanisms.

To summarize, we have shown for the first time that in human GFBLs, Cx43 not only assembles into GJ plaques but also forms HC plaques *in vitro* and *in vivo*. In cultured GFBLs, selective blockage of Cx43 HCs modulates the expression of key wound healing-associated genes through suppression of ATP release and activation of the ERK1/2 signaling pathway.

## Materials and Methods

### Tissue Samples

To obtain gingival tissue samples from three healthy individuals (26- and 27-year-old females and a 48-year-old-male), standardized, full-thickness excisional biopsies (2 × 10 mm) were collected under local anesthesia from healthy palatal attached gingiva in an area between the canine and the third molar using a double-bladed scalpel. Samples were processed for frozen sectioning as described previously^37^. For the study, a minimum of three tissue sections from each of the three subjects was analyzed.

### Cell Culture

Three human gingival fibroblast strains (GFBLs; GFBL-OL, GFBL-DC, and GFBL-HN) were isolated from clinically healthy attached gingiva from healthy 30 and 41-year-old male and 18-year-old female donors, respectively, as previously described^78^. These cell lines have been extensively characterized previously^37,79^. These fibroblast strains express Cx43 as their main GJ protein^37^. Cells were routinely maintained in Dulbecco’s Modified Eagle’s medium (DMEM), supplemented with 1% antibiotic/antimycotic and 10% fetal bovine serum (FBS) (Gibco Life Technologies, Inc., Grand Island, NY, USA) at 37 °C and 5% CO_2_, and seeded for experiments when they reached about 95% confluence. For high-density cultures, cells were seeded at a density of 42,000 cells/cm^2^, and for low-density cultures at 4,200 cells/cm^2^. Experiments were performed at passages 5 to 10.

### Ethics Statement

Gingival tissue donors provided written informed consent. Procedures were reviewed and approved by the Office of Research Ethics of the University of British Columbia, and comply with the ethical rules for human experimentation that are stated in the 1975 Declaration of Helsinki.

### Immunostaining

Human gingival frozen tissue sections and the fibroblast cultures were fixed and stained as described previously^37^. In order to investigate the localization of total Cx43, a polyclonal antibody against the cytoplasmic domain of Cx43 that recognizes intracellular, GJ-, and HC-associated Cx43 (total Cx43) was used (Supplementary Table S1)^39,49^. To localize Cx43 HCs, immunostaining was performed with an affinity-purified rabbit antibody Cx43(E2) that specifically targets the E2 loop domain of Cx43 and also blocks its HC function without affecting GJs (Supplementary Table S1)^41,42^. Localization of Cx43 intracellularly and on cell membranes was assessed with treatment of fixed cell with or without Triton X-100, respectively, before immunostaining. Images were acquired using optical sectioning at 1 μm (ECLIPSE 80i Microscope; Nikon, Tokyo, Japan), and are presented as z-stacks created by the NIS-Elements BR software (Nikon). Control stainings were performed by omitting the primary antibodies used in the study.

### Modulation of Cx43 GJ and HC Function

To study Cx43 function, fibroblasts were seeded on 6-well plates in their normal growth medium as above. After 48 h, cells were serum-starved for 24 h, and then treated with Cx43 mimetic peptide Gap27 (150 μM; SRPTEKTIFII; Biomatik, Cambridge, ON, Canada) that corresponds to the second extracellular (E2) loop domain of Cx43, and blocks its GJ and HC functions^38,51,52^, and Gap19 (250 and 400 μM; KQIEIKKFK; LifeTein, Hillsborough, NJ, USA) or TAT-Gap19 peptide (200, 400, 500, and 600 μM; YGRKKRRQRRR-KQIEIKKFK; LifeTein) that interacts with nine amino acids in the LT-domain of the cytoplasmic loop of Cx43 and specifically blocks its HC function without affecting GJs^53,54^. Control samples were treated with scrambled control Gap27 peptide (TFEPDRISITK; Biomatik)^80^, or mutated, function-deficient control TAT-Gap19 peptide (YGRKKRRQRRR-KQAEIKKFK; LeifTein)^54^, respectively.

### Quantitative Real-Time RT-PCR (qPCR)

qPCR analysis was performed according to MIQE guidelines^81^ as we have described in detail previously^37^. The primers used for qPCR and reference genes are listed in Supplementary Table S2. Amplification reactions for qPCR were performed using the CFX96 System (Bio-Rad). For a given experiment, at least two reference genes were chosen^82^. Non-transcribed RNA samples were used as a negative control. The qPCR reactions were performed in triplicate for each sample. The data was analyzed and is presented based on the comparative Ct method (CFX Manager Software Version 2.1, Bio-Rad).

### Preparation of Cell Lysates for Western Blotting

To collect cell lysates, cells were washed with ice-cold phosphate-buffered saline (PBS), and lysed with a buffer containing 25 mM Tris-HCL (pH 7.6), 100 mM Octyl β-D-glucopyranoside, 5 mM NaF, 1 mM Na3VO4 (Sigma-Aldrich, St. Louis, MO, USA), and the Complete Protease Inhibitor Cocktail (Roche Diagnostics, Laval, Quebec, Canada), dissolved in H_2_O. Lysates were collected using a rubber policeman, and filtered through a NucleoSpin Filter (Macherey-Nagel) by centrifugation at 5,000 g for 10 min.

To assess the distribution of Cx43 in different cellular fractions, cell lysates were obtained by sequential treatment with 1% Triton X-100 (representing non-lipid raft- associated and intracellular pool) followed by a treatment with octyl β-D-glucopyranoside containing buffer (representing Triton X-100 insoluble lipid raft-associated pool) as above^44,47^.

### Western Blotting

The activation of the ERK1/2 signaling pathway by Cx43 mimetic peptides or apyrase (Sigma-Aldrich), which selectively degrades extracellular ATP^58,59^, was studied by Western blotting as described previously^37^ using cell lysates obtained as described above. For the experiments, GFBLs were seeded on 6-well plates, treated with Gap27 (150 µM), TAT-Gap19 (400 µM), and apyrase (1 U/mL), or equal amount of control peptides and vehicle control (dH_2_O) for 1, 2, 6, and 24 h, and cell lysates collected as above. Western blotting was performed with antibodies against total or phosphorylated forms of the ERK1/2 pathway (Supplementary Table S1). β-Tubulin was used as a loading control.

### Blocking of ERK1/2 and ATP Signaling Pathways

To determine the role of the ERK1/2 signaling pathway in Cx43 mimetic peptide-induced gene expression, we blocked this pathway by MEK1/2 inhibitor PD184352 (10 μM; Sigma-Aldrich) in Gap27- or TAT-Gap19-treated cells, respectively. To this end, confluent GFBL cultures were pre-incubated with PD184352 at 37 ^o^C for 1 h, and then treated with Gap27 (150 μM) or TAT-Gap19 (400 μM) with PD184352 in serum-free growth medium for 24 h. PD184352 was dissolved in DMSO, and control samples were treated with respective amounts of DMSO only. Total RNA was collected for qPCR as described above.

To study the role of the ATP signaling pathway in Cx43 HC-regulated gene expression, cells were cultured in high density in their normal growth medium, and serum-starved as above, and then treated with apyrase (1 U/mL) or vehicle control (dH_2_O) in serum-free growth medium for 24 h. Total RNA was then collected for qPCR as described above.

### Dye Transfer Experiments

To assess the GJ and HC functions of Cx43, dye transfer assays were performed^49^. To this end, fibroblast cultures were generated on gelatin-coated glass coverslips in 24-well plates as described previously^37^. To assess dye transfer through GJs by scrape loading, cells were seeded on the coverslips in their normal growth medium as described above and then serum-starved in DMEM for 24 h, followed by pre-incubation with Gap27 (150 μM), TAT-Gap19 (400 μM), Cx43(E2) antibody (1 mg/mL), meclofenamic acid (MFA; 50 μM; Sigma-Aldrich), a widely used GJ inhibitor^50^, or with corresponding peptide, non-immune rabbit IgG, or vehicle controls in DMEM at 37 ^o^C for 1 h. Medium was then removed and a scrape wound was created through the cell layer with a 10-μL pipette tip, and cells incubated as above with 0.5% Lucifer Yellow (Molecular Probes Inc., Eugene, OR, USA) in PBS+ for 5 min at 37 °C. Cells were then rinsed and fixed as described previously^37^.

To assess the HC function of Cx43, cells were cultured on gelatin-coated glass coverslips and serum-starved for 24 h as above. Cells were then preincubated in their normal growth medium (DMEM) that contains 1.8 mM Ca^2+^, or in EMEM (Lonza, Walkersville, MD, USA) supplemented with 180 nM Ca^2+^ (low calcium medium), which induces the opening of Cx HCs^38^, and treated with Gap27, TAT-Gap19, and Cx43(E2) antibody, or corresponding controls, as above for 1 h, followed by incubation in the respective media with the inhibitors or controls and Propidium Iodide (2.5 mM; Sigma-Aldrich) for 20 min. After incubation, media was removed and cells were rinsed with PBS+, and fixed as described previously^37^.

### Statistical Analysis

The data is presented as mean +/− standard error of the mean (s.e.m.) from a minimum of three biological replicates, unless otherwise indicated. Statistical analysis was performed by using two-tailed *t*-test; p < 0.05 was considered statistically significant. Values obtained from the qPCR by the comparative Ct-method were Log2 transformed for statistical testing^83^.

### Data availability

All data generated or analyzed during this study are included in this published article (and its Supplementary Information files).

## Electronic supplementary material

Supplementary Information
