# Supplementary material for: Connexin 43 Hemichannels Regulate the Expression of Wound Healing-Associated Genes in Human Gingival Fibroblasts
Source: Sci Rep. 2017 Oct 26;7:14157. doi: 10.1038/s41598-017-12672-1 (PMC5658368; doi:10.1038/s41598-017-12672-1)
Supplement: Supplementary file 1 — Supplementary Information [file 41598_2017_12672_MOESM1_ESM.doc]

**Connexin 43 Hemichannels Regulate the Expression of Wound Healing-Associated Genes in Human Gingival Fibroblasts**

Rana Tarzemany^1^, Guoqiao Jiang^1^, Jean X. Jiang^2^, Hannu Larjava^1^, Lari Häkkinen^1,*^

^1^Department of Oral Biological and Medical Sciences, Faculty of Dentistry, The University of British Columbia, Vancouver, BC, Canada V6T 1Z3

^2^Department of Biochemistry, University of Texas Health Science Center, San Antonio, Texas, USA 78229-3900

***Corresponding Author:** Dr. Lari Häkkinen, The University of British Columbia, Faculty of Dentistry, Department of Oral Biological and Medical Sciences, 2199 Wesbrook Mall, Vancouver, BC, Canada V6T 1Z3 Canada; E-mail: [lhakkine@dentistry.ubc.ca](mailto:lhakkine@dentistry.ubc.ca); Tel: +604-822-0096.

**Supplementary Figure S1. The expression, abundance and distribution of Cx43 in high- and low-density cultures of human gingival fibroblasts.** (A and B) Representative phase contrast images from human gingival fibroblasts (GFBL-DC) cultured at high-density (HD; 100% confluence, A) and low-density (LD; 10% confluence, B) conditions. Immunolocalization of Cx43-positive plaques in HD and LD cultures detected with an antibody that recognizes all Cx43 molecules (C, D, G and H), and with the Cx43 HC-specific Cx43(E2) antibody (E, F, I and J). (C-F) Cells were fixed and permeabilized with 0.5% Triton X-100 treatment before immunostaining. In HD (C) and LD (D), total Cx43 staining localized abundantly throughout the cells. Cx43 HC plaques were markedly smaller than total Cx43 staining in both HD (C and E, respectively) and LD (D and F, respectively) cultures. In LD cultures, no Cx43 HC-specific immunoreactivity was noted in the long cell extensions (insert in F), while they contained plaques that were detected with the antibody against total Cx43 (insert in D). (G-J) Immunolocalization of Cx43-positive plaques with the total Cx43 (G and H) or Cx43(E2) antibody (I and J) in HD (G and I, respectively) and LD (H and J, respectively) cultures pretreated with 1% Triton X-100 for 10 min before fixation and immunostaining as above. In general, Triton X-100 pretreatment resulted in significant reduction in the amount of small Cx43-positive plaques (likely representing detergent soluble non-lipid raft and intracellular Cx43) as compared to non-pretreated samples (G-J and C-F, respectively). Immunostaining of the Triton X-100 insoluble fractions with the Cx43 antibody in HD (G) showed presence of large Cx43-positive plaques, likely representing lipid raft-associated Cx43 gap junction plaques (GJs), while the number of these plaques was markedly reduced in LD cultures (H). Immunostaining of the Triton X-100 insoluble fractions with the Cx43(E2) antibody in HD (I) and in LD (J) cultures revealed presence of few small- sized Cx43 non-lipid raft associated plaques in detergent insoluble fractions. Magnification bars = 10 μm. (K) qPCR analysis of HD and LD cultures showed similar relative amount of Cx43 mRNA in both conditions. (L and M) Similarly, Western blotting showed that the total Cx43 protein abundance per cell was not affected by cell density. However, cells in HD cultures showed significantly higher levels of Cx43 phosphorylated at S262 (P2), and S279/282 and S256 (P1), previously associated with Cx43 present in GJs [39,40], as compared to LD cultures. In contrast, LD cultures had a significantly higher level of Cx43 phosphorylated at S368 (P0), corresponding to the previously described non-junctional (HC and intracellular) Cx43 pool [39,40]. Sample loading was normalized for β-Tubulin levels. Results show mean +/- s.e.m from three independent experiments. Statistical analysis was performed by Student’s t-test (*p<0.05, **p<0.01). (N) Western blotting analysis of distribution of Cx43 in 1% Triton X-100 (TX-100) insoluble (lipid raft-associated Cx43) and soluble (non-lipid raft and intracellular Cx43) fractions in HD cultures. Majority of Cx43 phosphorylated forms (P1 and P2) were associated with the Triton X-100 insoluble (lipid raft-associated) pool, while Triton X-100 soluble Cx43 was predominantly non-phosphorylated (P0). For adequate signal detection, sample loading in detergent soluble and insoluble fractions was increased relative to total.

**Supplementary Figure S1.**


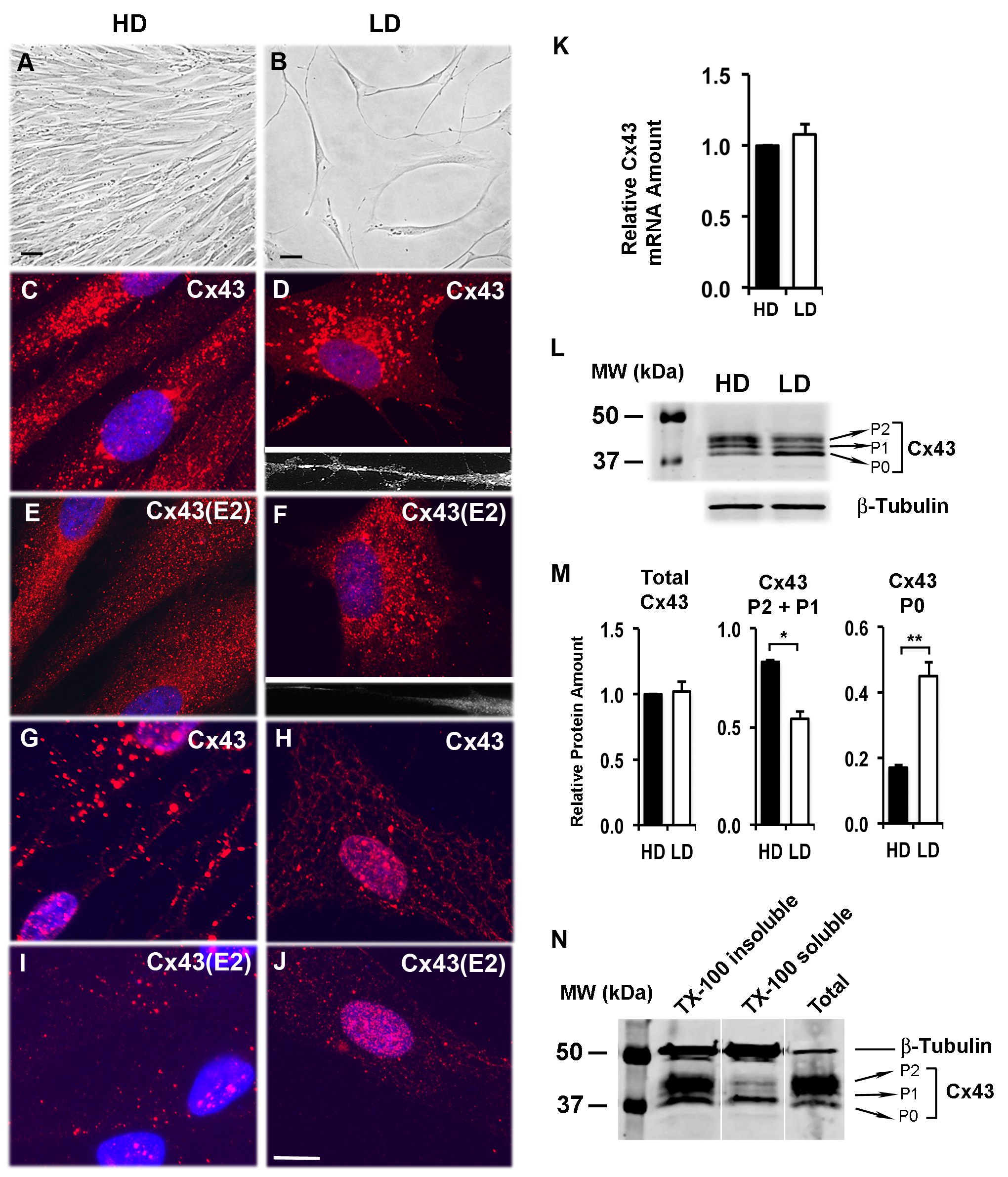


**Supplementary Figure S2.** **The** **expression of a set of genes in human gingival fibroblasts treated with increasing concentrations of TAT-Gap19 or Gap19 relative to control samples.** Confluent GFBL-DC cultures were treated with (A) increasing concentrations of TAT-Gap19 (200, 400 and 500 μM) or control peptide (200, 400 and 500 μM), and (B) increasing concentrations of Gap19 (250 and 400 μM) or control peptide (250 and 400 μM) for 24 h, and expression of a set of genes involved in wound healing was analyzed by qPCR. Results represent mean +/- s.e.m. of mRNA amount relative to control peptide-treated cells from two repeated experiments. TN-C: Tenascin-C.

S**upplementary Figure S2.**

**
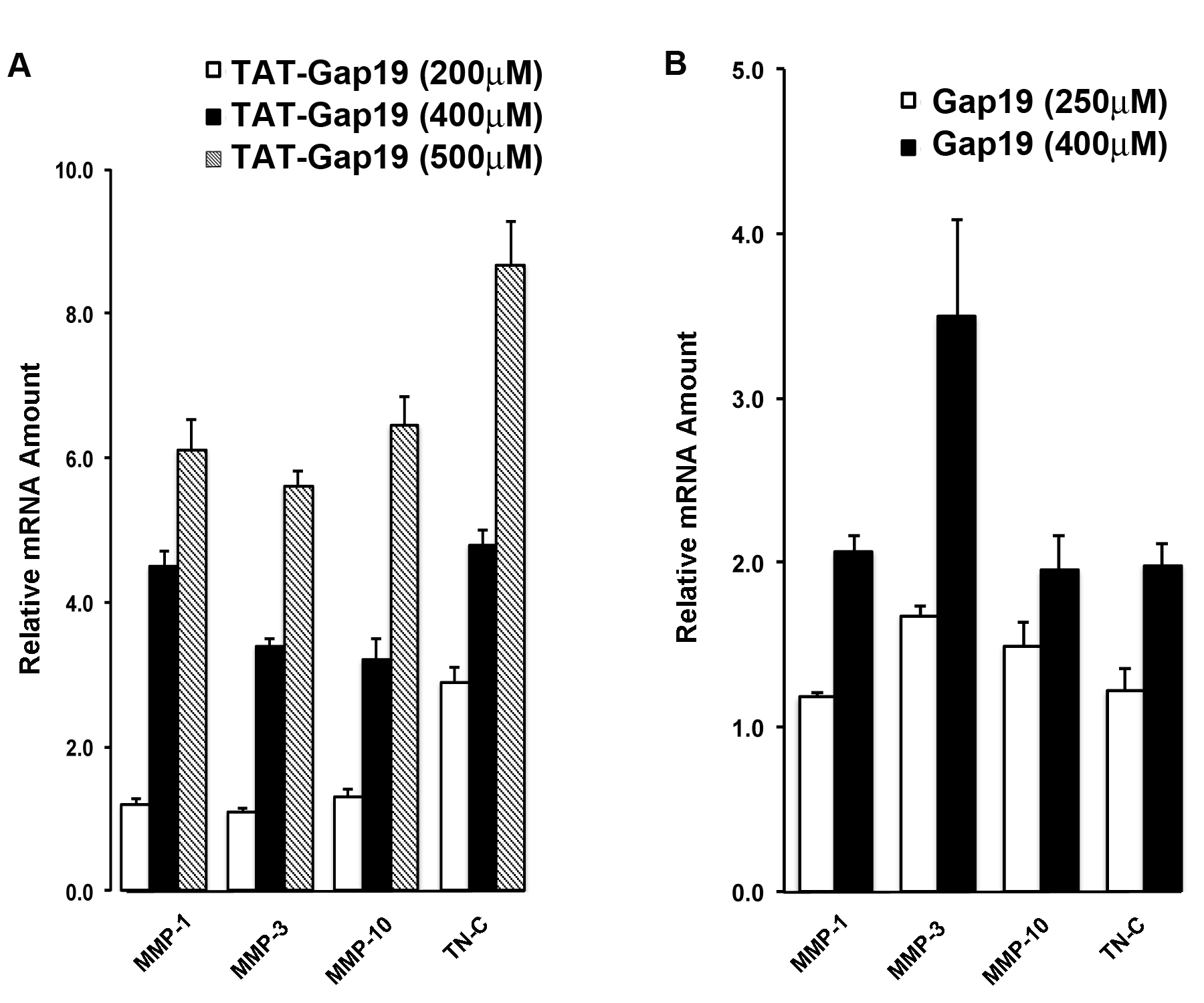
**

**Supplementary Figure S3.** **The** **expression of a set of genes in human gingival fibroblasts treated with TAT-Gap19 with or without apyrase relative to control samples.** Confluent GFBL-DC cultures were treated with TAT-Gap19 (400 μM) with or without apyrase (1 U/mL) for 24 h, and expression of a set of genes involved in wound healing was analyzed by qPCR. Results represent mean +/- s.e.m of mRNA amount relative to control peptide/vehicle treated cells from three parallel samples from one experiment. TN-C: Tenascin-C; α-SMA: α-Smooth Muscle Actin; VEGF-A: Vascular Endothelial Growth Factor-A.

**Supplementary Figure S3.**

**
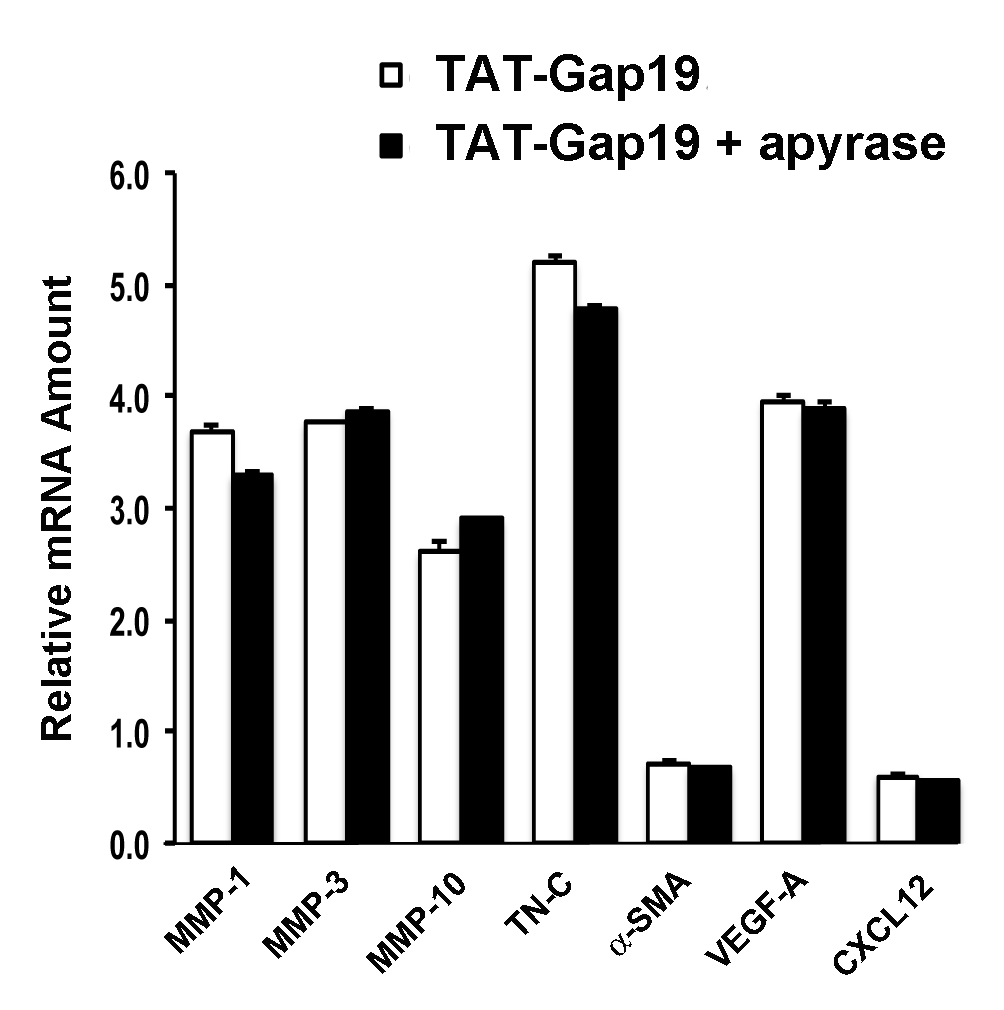
**

**Supplementary Figure S4. The** **expression of a set of ATP and adenosine receptor signaling genes in human gingival fibroblasts treated with Gap27 or TAT-Gap19 relative to control samples.** Confluent cultures of GFBL-DC were treated with Gap27 (150 μM) or TAT-Gap19 (400 μM) for 24 h, and amount of mRNA for set of ATP and adenosine receptor genes was analyzed by qPCR. Results represent mean +/- s.e.m. of relative amount of mRNA from a minimum of three repeated experiments (**p<0.01, ***p<0.001; two-tailed *t*-test). ADA: adenosine deaminase.

**Supplementary Figure S4.**

**
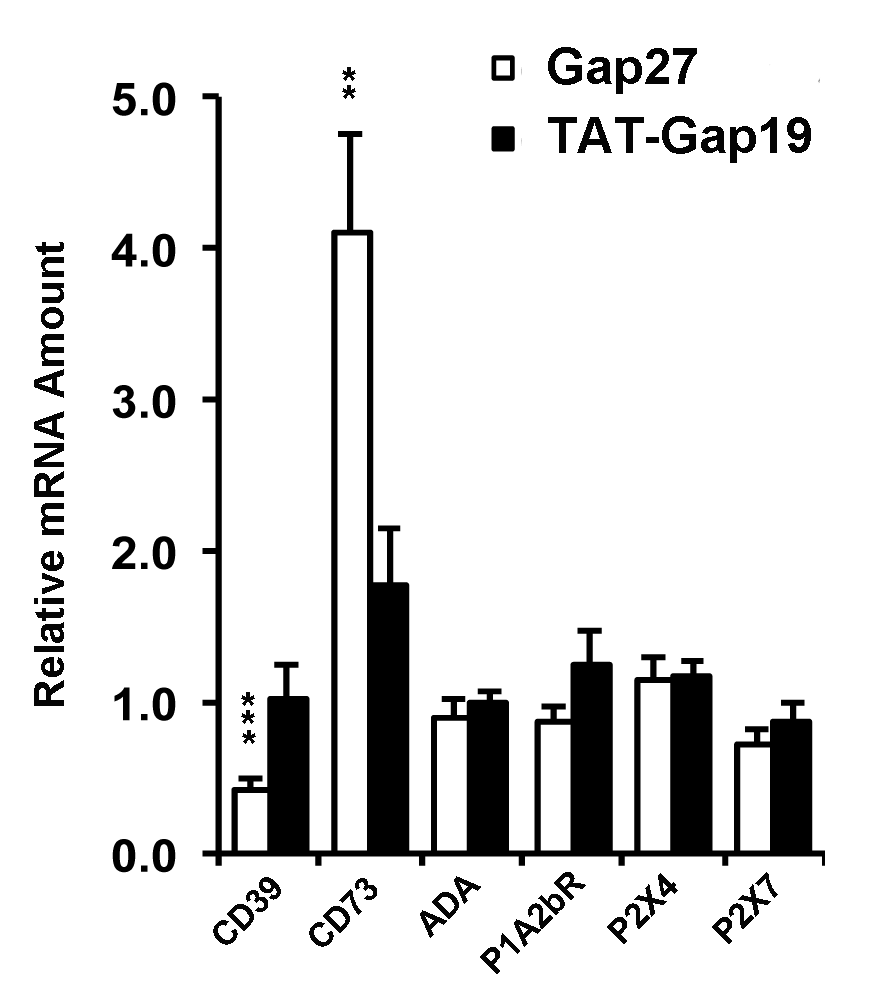
**

**Supplementary Figure S5. Full-length images of Western blotting analysis of activation of ERK1/2 pathway by Gap27, TAT-Gap19, and apyrase treatment in gingival fibroblasts.** Results show Western blotting analysis of ERK1/2 pathway activation in confluent GFBL-DC cultures that were treated with Gap27 or control peptide (150 μM), TAT-Gap19 or control peptide (400 μM), and (apyrase (1 U/mL) or vehicle control (dH_2_O) for 1, 2, 6, and 24 h.

**Supplementary Figure S5.**

**
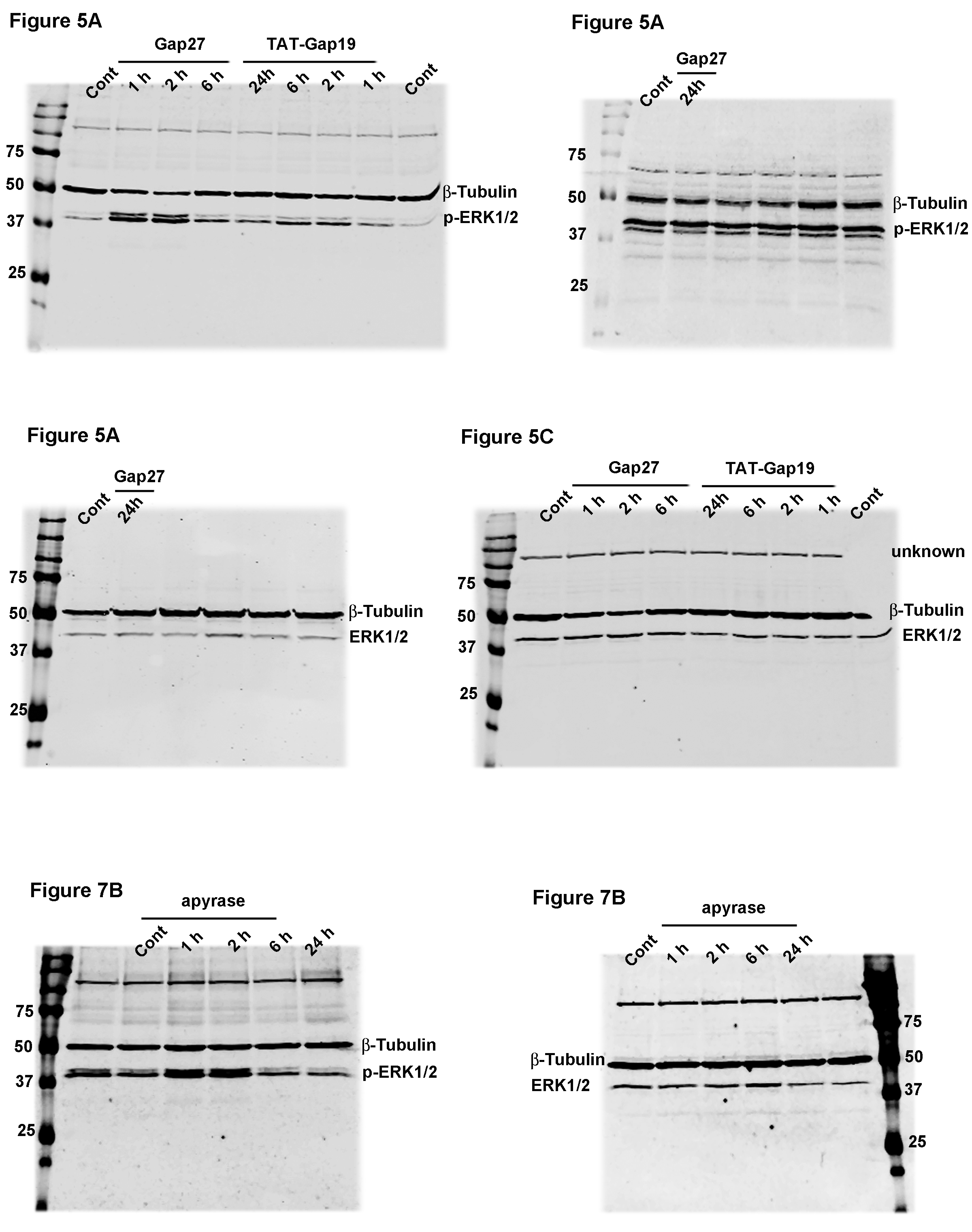
**

**Supplementary Table S1. List of antibodies used for immunostaining and Western blotting.**

| **Antibody** | **Manufacturer** | **Source** | **Dilution** | |
| --- | --- | --- | --- | --- |
|  |  |  | **Immunostaining** | **Western blotting** |
| Anti-Vimentin  (M7020; Vim 3B4) | DakoCytomation,  Burlington, ON, CA | Mouse | 1:200 |  |
| Anti-Connexin 43  (C6219) | Sigma-Aldrich, St. Louis, MO, USA | Rabbit | 1:800 | 1:8000 |
| Cx43(E2) | Kindly provided by Dr. Jean X. Jiang, University of Texas Health Science Center, San Antonio, TX, USA | Rabbit | 1:300 |  |
| Anti-ERK1  (ab7947) | Abcam Inc. Cambridge, MA, USA | Rabbit |  | 1:500 |
| Anti-active MAPK  (pTEpY) | Promega, Madison, WI, USA | Rabbit |  | 1:2000 |
| Anti-β-Tubulin (ab21057) | Abcam Inc. | Goat |  | 1:1000 |

**Supplementary Table S2. Primers used for quantitative real-time RT-PCR.**

| **GeneBank** | **Gene** | **Primer sequence** | **Orientation** | **Location** | **Amplicon (bp)** |
| --- | --- | --- | --- | --- | --- |
| **MMPs and TIMPs** | | | | | |
| NM_002421 | MMP-1 | GCTAACAAATACTGGAGGTATGATG | Forward | 1250-1275 | 100 |
|  |  | GTCATGTGCTATCATTTTGGGA | Reverse | 1304-1325 |  |
| NM_001166308 | MMP-3 | ATGATGAACAATGGACAAAGGA | Forward | 661-682 | 91 |
|  |  | GAGTGAAAGAGACCCAGGGA | Reverse | 751-732 |  |
| NM_002425 | MMP-10 | TTATACACCAGATTTGCCAAGA | Forward | 394-415 | 56 |
|  |  | TTCAGAGCTTTCTCAATGG | Reverse | 450-432 |  |
| NM_004995 | MMP-14 | TCTCCCAGAGGGTCATTCAT | Forward | 618-1637 | 70 |
|  |  | TTCCAGTATTTGTTCCCCTTGTAG | Reverse | 1688-1665 |  |
| NM_003254 | TIMP-1 | CTGTGTCCCACCCCACC | Forward | 267-283 | 64 |
|  |  | GAACTTGGCCCTGATGACGA | Reverse | 330-311 |  |
| NM_003255 | TIMP-2 | ACATTTATGGCAACCCTATCAA | Forward | 481-502 | 70 |
|  |  | TCAGGCCCTTTGAACATCTTTA | Reverse | 550-529 |  |
| NM_000362 | TIMP-3 | AGGACGCCTTCTGCAAC | Forward | 1281-1297 | 68 |
|  |  | CTCCTTTACCAGCTTCTTCC | Reverse | 1348-1329 |  |
| NM_003256 | TIMP-4 | ACCTGTCCTTGGTGCAGA | Forward | 927-944 | 80 |
|  |  | TGTAGCAGGTGGTGATTTGG | Reverse | 1004-985 |  |
| **Fibrillar ECM proteins** | | | | | |
| BC036531 | Collagen type I (alpha 1) | AACCAAGGCTGCAACCTGGA | Forward | 3951-3970 | 80 |
|  |  | GGCTGAGTAGGGTACACGCAGG | Reverse | 4030-4009 |  |
| NM_000090 | Collagen type III (alpha 1) | CTCCTGGGATTAATGGTAGT | Forward | 1271-1290 | 70 |
|  |  | CCAGGAGCTCCAGGAAT | Reverse | 1340-1324 |  |
| NM_212482 | EDA-FN (Extra Domain A-Fibronectin) | CACAGTCAGTGTGGTTGCCT | Forward | 5633-5652 | 68 |
|  |  | CTGTGGACTGGGTTCCAATCA | Reverse | 5700-5680 |  |
| NM_212482 | EDB-FN (Extra Domain B-Fibronectin) | CAGTAGTTGCGGCAGGAGAA | Forward | 4168-4188 | 65 |
|  |  | GTATCCTACTGAGGAGTCCACAAAATC | Reverse | 4232-4206 |  |
| **Matricellular proteins** | | | | | |
| NM_002160 | TN-C  (Tenascin-C) | CAACCTGATGGGGAGATATGGGGA | Forward | 6769-6792 | 75 |
|  |  | GAGTGTTCGTGGCCCTTCCAG | Reverse | 6846-6826 |  |
| **Contractility and myofibroblast associated proteins** | | | | | |
| NM_001613 | α –SMA  (α-Smooth Muscle Actin) | AGCGTGGCTATTCCTTCGT | Forward | 637-655 | 97 |
|  |  | CTCATTTTCAAAGTCCAGAGCTACA | Reverse | 733-707 |  |
| NM_005964 | NMMIIB (Non-Muscle Myosin IIB) | CCGTTTTACATAATCTGAAGGATC | Forward | 395-418 | 98 |
|  |  | TTGGAAGATTCTTGTAAGGGTT | Reverse | 493-472 |  |
| **Small leucine-rich proteoglycans** | | | | | |
| BT019800 | DCN  (Decorin) | CTGACACAACTCTGCTAGAC | Forward | 242-261 | 97 |
|  |  | GACAAGAATCAATGCGTGAAG | Reverse | 339-319 |  |
| NM_002023 | FMOD (Fibromodulin) | CACAATGAGATCCAGGAAG | Forward | 761-779 | 85 |
|  |  | TCCGAAGGTGGTTATAACTC | Reverse | 845-826 |  |
| **TGF-β signaling related genes** | | | | | |
| NM_000660 | TGF-β1 | CAACGAAATCTATGACAAGTTCAAGCAG | Forward | 1218-1245 | 76 |
|  |  | CTTCTCGGAGCTCTGATGTG | Reverse | 1294-1275 |  |
| NM_003239 | TGF-β3 | ACACCAATTACTGCTTCCGCAA | Forward | 1161-1182 | 81 |
|  |  | GCCTAGATCCTGTCGGAAGTC | Reverse | 1242-1220 |  |
| NM_005966 | NAB1  (NGFI-A Binding Protein-1) | CAAAGTCCCACTCATCAGAGA | Forward | 1930-1950 | 114 |
|  |  | TCACAGCTATCTTGAATCTTCAG | Reverse | 2043-2020 |  |
| **Growth factors and cytokines** | | | | | |
| NM_001171630 | VEGF-A (Vascular Endothelial Growth Factor-A) | AGTGTGTGCCCACTGAGGA | Forward | 1316-1334 | 97 |
|  |  | GTGCTGTAGGAAGCTCATCTC | Reverse | 1413-1393 |  |
| NM_199168 | CXCL12/SDF-1α | TACAGATGCCCATGCCGA | Forward | 174-191 | 93 |
|  |  | CTGAAGGGCACAGTTTGGAG | Reverse | 266-247 |  |
| **Cell-cell junction proteins** | | | | | |
| NM_000165 | Cx43 | AGCAGTCTGCCTTTCGTTGTA | Forward | 393-412 | 73 |
|  |  | GATTGGGAAAGACTTGTCATAGCAG | Reverse | 466-442 |  |
| NM_001097519 | Cx45 | AGCTGGGTCCAACAAAAGC | Forward | 1151-1169 | 108 |
|  |  | ACCATAAACTATGAGAAGCACAGATT | Reverse | 1258-1233 |  |
| NM_001792 | Cadherin-2 | TGAGGAGTCAGTGAAGGAG | Forward | 843-861 | 91 |
|  |  | CTTCTGCCTTTGTAGGTGG | Reverse | 933-915 |  |
| **ATP and adenosine signaling** | | | | | |
| NM_001776 | CD39 | CCTCTATGGCAAGGACTACA | Forward | 1044~1063 | 133 |
|  |  | ATGAAAGCATGGGTCCCT | Reverse | 1177~1159 |  |
| NM_001204813 | CD73 | AGCATTCCTGAAGATCCAAGC | Forward | 1493~1513 | 149 |
|  |  | GTTGCCCATGTTGCATTCTC | Reverse | 1642~1623 |  |
| NM_000022 | ADA (Adenosine deaminase) | CACCCTGGACACTGATTACC | Forward | 1034~1053 | 149 |
|  |  | CCATAGGCTTTATAGAGCAGGT | Reverse | 1183~1162 |  |
| NM_000674 | P1A1R | CTACATTGCCATCTTCCTCAC | Forward | 1243~1263 | 111 |
|  |  | GAAATGGTCATTCCAAATCTTAAGG | Reverse | 1354~1330 |  |
| NM_000675 | P1A2aR | TCGGTTGTGAATCCCTTCATCTA | Forward | 1300~1321 | 111 |
|  |  | CAGCTGCCTTGAAAGGTTCTT | Reverse | 1411~1391 |  |
| NM_000676 | P1A2bR | CTGCCTCTCTTGAGCACTTC | Forward | 1437~1456 | 134 |
|  |  | GCTGTTGGCATAATCCACAC | Reverse | 1571~1552 |  |
| NM_000677 | P1A3R | GCTTGTGTGGTCTGCCATC | Forward | 1662~1680 | 114 |
|  |  | TGTTGATGGGGAATCTGAAGG | Reverse | 1776~1756 |  |
| NM_002563 | P2Y1 | GACAAGTGTTCACTCACATCTG | Forward | 2803~2824 | 154 |
|  |  | TGTGGATGACTGTCAACCTC | Reverse | 2957~2938 |  |
| NM_002564 | P2Y2 | TGAGCACAGAGAAAAGTCAGG | Forward | 2318~2338 | 91 |
|  |  | AATGGCTTAACATTTACCAGCA | Reverse | 2409~2388 |  |
| NM_002560 | P2X4 | ACAACATCTGGTATCCCAAA | Forward | 876~895 | 141 |
|  |  | TTCTCCACTATTTTGCCAAGA | Reverse | 1017~997 |  |
| NM_002562 | P2X7 | CAGCCCTGTGTGGTCAAC | Forward | 1266~1283 | 143 |
|  |  | TTGCAGACTTCTCCCTAGTAGC | Reverse | 1409~1388 |  |
| **Cell cycle** | | | | | |
| NM_001237 | Cyclin A2 | TCACTAACAGTATGAGAGCTATCC | Forward | 922-945 | 128 |
|  |  | GCACTGACATGGAAGACAG | Reverse | 1050-1032 |  |
| NM_031966 | Cyclin B1 | TGTGTCAGGCTTTCTCTGA | Forward | 678-696 | 121 |
|  |  | CTCAAGTTGTCTCAGATAAGCA | Reverse | 799-778 |  |
| NM_053056 | Cyclin D1 | GGAGAGGATTAGGTTCCATCC | Forward | 1479-1499 | 137 |
|  |  | TCAGGAAAAGCACAAGAATATGT | Reverse | 1616-1594 |  |
| NM_001322262 | Cyclin E1 | GCTGGGCAAATAGAGAGGA | Forward | 462-480 | 136 |
|  |  | CTCCATTAACCAATCCAGAAGAA | Reverse | 598-576 |  |
| **Reference genes** | | | | | |
| NM_002046 | GAPDH (Glyceraldehydes-3-phosphate d­dehydrogenase) | CTTTGTCAAGCTCATTTCCTGGTA | Forward | 1020-1043 | 70 |
|  |  | GGCCATGAGGTCCACCA | Reverse | 1089-1073 |  |
| M31642 | HPRT1 (Hypoxanthine phosphoribosyltransferase I) | TGTTGGATTTGAAATTCCAGACAAG | Forward | 619-643 | 107 |
|  |  | CTTTTCCAGTTTCACTAATGACACAA | Reverse | 727-700 |  |
| NM_021009 | UBC  (Ubiquitin C) | GTGGCACAGCTAGTTCCGT | Forward | 371-389 | 96 |
|  |  | CTTCACGAAGATCTGCATTGTCA | Reverse | 444-467 |  |
| NM_004048 | B2M (Beta-2-microglobulin) | TGTCTTTCAGCAAGGACTGGTCTTTC | Forward | 281-306 | 92 |
|  |  | ATGGTTCACACGGCAGGCATA | Reverse | 351-372 |  |
| NM_001172085 | TBP  (TATAA-box binding protein) | TTCGGAGAGTTCTGGGATTG | Forward | 542-562 | 94 |
|  |  | ACGAAGTGCAATGGTCTTTAG | Reverse | 635-614 |  |
